# Postnatal depression and its association with adverse infant health outcomes in low- and middle-income countries: a systematic review and meta-analysis

**DOI:** 10.1186/s12884-020-03092-7

**Published:** 2020-07-22

**Authors:** Abel Fekadu Dadi, Emma R. Miller, Lillian Mwanri

**Affiliations:** 1 Institute of Public Health, College of Medicine and Health Sciences, University of Gondar, Gondar, Ethiopia; 2 College of Medicine and Public Health, Flinders University, Health Sciences Building, Sturt Road, Bedford Park, Adelaide, SA 5001 Australia

**Keywords:** Postnatal depression, Adverse infant health outcomes, Systematic review, Meta-analysis, Low, And middle-income countries

## Abstract

**Background:**

Postnatal Depression (PND) is a mood disorder that steals motherhood and affects the health and development of a newborn. While the impact of PND on motherhood and newborn in developed countries are well described, its epidemiology and health consequences in infant is not well known in middle-and low-income countries. The objective of this review was to determine the burden and association of PND with adverse infant health outcomes in low-and middle- income countries.

**Methods:**

We searched observational studies written in the English language and conducted in middle-and low-income countries between December 1st, 2007, and December 31st, 2017. The CINHAL, MEDLINE, Emcare, PubMed, Psych Info, and Scopus databases were searched for the following search terms: PND, acute respiratory infection, pneumonia, diarrhea, exclusive breastfeeding, common infant illnesses, and malnutrition. We excluded studies in which the primary outcomes were not measured following a standardized approach. We have meta-analyzed the estimates from primary studies by adjusting for possible publication bias and heterogeneity. The analysis was conducted in Stata 14. The study was registered in PROSPERO protocol number CRD42017082624.

**Result:**

Fifty-eight studies on PND prevalence (among 63,293 women) and 17 studies (among 32,454 infants) on infant health outcomes were included. PND prevalence was higher in the low-income countries (Pooled prevalence (PP) = 25.8%; 95%CI: 17.9–33.8%) than in the middle-income countries (PP = 20.8%; 95%CI: 18.4–23.1%) and reached its peak in five to ten weeks after birth. Poor obstetric history and social support, low economic and educational status, and history of exposure to violence were associated with an increased risk of PND. The risk of having adverse infant health outcomes was 31% higher among depressed compared to non-depressed postnatal mothers (Pooled relative risk (PRR) = 1.31; 95%CI: 1.17–1.48). Malnutrition (1.39; 1.21–1.61), non-exclusive breastfeeding (1.55; 1.39–1.74), and common infant illnesses (2.55; 1.41–4.61) were the main adverse health outcomes identified.

**Conclusions:**

One in four and one in five postnatal mothers were depressed in low and middle-income countries, respectively. Causes of depression could be explained by social, maternal, and psychological constructs. High risk of adverse infant health outcomes was associated with PND. Timely screening of PND and evidence-based interventions were a pressing need in low and middle-income countries.

## Background

Worldwide, depression is the most common mental illness and the leading cause of maternal morbidity and disability in the perinatal period [1, 2]. Postnatal depression (PND) is a crippling mood disorder that steals motherhood [3]. It is characterized by signs and symptoms such as low mood, tiredness, insomnia, irritability, and reduced functioning [4]. The symptoms of PND commences four to six weeks after childbirth [4] and get peak two to three months after birth [3, 5, 6]. The causes of PND are explained by genetics and socio-environmental constructs [7].

Postnatal depression is used to be an issue of Western countries [8], and research focus on this problem has been limited until the relationship found between socioeconomic status and PND [9, 10]. Recent evidence has shown that the prevalence of PND is higher in low-and middle-income countries (range from 7 to 33%) than high-income countries (range between 13 and 19%) [5, 11, 12]. Postnatal depression found to be heterogeneous in Africa with lower prevalence in Uganda (7.1%) and the highest in Zimbabwe (33%) [13]. Far less is known about PND, although more than 90% of the world’s children are living in low-and middle-income countries [13]. This suggests more concise and updated estimate about PND epidemiology and consequences are pivotal.

Postnatal depression is the second cause of disability next to HIV/AIDS [2], and the most known complication of childbirth [14]. Postnatal depression can increase the cost of the health care system [15], reduce mother’s quality of life and workforce in the economy [16]. Postnatally depressed women are at risk of back pain, insomnia, thought of self-harm, suicidal ideation, and poor parenting behavior [17, 18]. Postnatal depression affects initiation of breastfeeding and effective utilization of available health services [17, 19]. This could lead to malnutrition and weakened immune systems with further predisposition to illnesses including diarrhea, pneumonia, measles, and other childhood illnesses [12, 20–22].

The identification of women potentially at risk of PND is vital to prevent the onset and subsequent consequences. The previous reviews on PND were descriptive, incomprehensive and inconclusive [11, 23–28], skewed to developed countries [4, 17, 23, 24], and lacked detail quantification of risk factors. The previous reviews about PND effects on adverse infant health outcomes were scarce, descriptive, outdated, and mostly included studies from developed countries [12, 13, 17]. We did this comprehensive review to explore the prevalence and thematically quantify and present most notable risk factors of PND, and to investigate its association with risk of adverse infant health outcomes in low- and middle-income countries.

## Methods

### Search strategy

We searched the CINHAL, MEDLINE, EMCare, PubMed, Psych Info, and Scopus databases for postnatal depression and its effect on adverse infant health outcomes. The following search terms were used: Postnat*, postpart*, depress*, exclusive breastfeeding, pneumonia, common infant illnesses, diarrhea, measles, diarrhea, fever, malnutrition, and infant feeding practices.

### Eligibility criteria

We included observational studies conducted in low and middle-income countries, written in the English language, and published between January 1st, 2007, and December 31st, 2017 with an aim of including only updated data on the topic. Furthermore, studies were included if they fulfilled the following main outcome definitions:- (1) malnutrition was measured using standard indices like wasting, stunting, short stature, or underweight/overweight; (2) age-related infant feeding practice that reported exclusive breastfeeding or complementary feeding; (3) common infant illnesses such as ARI (pneumonia, fever, and cough), malaria, measles, and diarrhea were assessed following the WHO Integrated Management of Newborn and Childhood Illnesses (IMNCI) guideline; (4) measurement of depression was done using a standard and validated screening tools. According to the World Bank Atlas, low-income and middle-income countries are those with the Gross National Income (GNI) per capita of ≤ $1025 and between $1026 to 12,375.

### Exclusion criteria

Studies that pooled antenatal and PND scores, had fair to poor quality score on the New Castle Ottawa Scale, studies restricted to very high or low-risk populations, conference proceedings, commentaries, abstracts, reports, and unpublished data were excluded.

### Data extraction and study quality assessment

This section of methodology has been published previously in recent paper [29].

### Data analysis

Estimates from primary studies were reported in prevalence, odds ratios, or relative risks. For the first objective, estimating the overall prevalence of PND, the prevalence extracted from all primary studies were meta-analyzed. For the second objective, identifying prominent risk factors of PND, odds ratios obtained for each risk factor identified from each primary study were meta-analyzed to get a pooled odds ratio for that specific risk factor. For the third objective, investigating the association between PND and adverse infant health outcomes, relative risk estimates obtained from each primary study were collected and meta-analyzed to get a single estimate for each adverse birth outcomes. The meta-analysis for each objective was reported in a separate forest plot, and their main findings were summarized in tables. For studies that lacked adjusted estimates, crude estimates were used. Where a single study reported more than one adverse birth outcomes, pooling was done for each outcome.

### Risk of bias and adjustment

Cochran Q (*I*^*2*^), visual inspection of forest plot, Galbraith plot [30], and Higgins test [31] were used to assess the presence of heterogeneity. The DerSimonian and Lairds random-effect model was used to pool odds ratio or relative risk estimates in the presence of heterogeneity. These sub-group analyses investigated differences between and within groups’ effect. Study setting, study design, year of investigation, tools used for measuring depression, sample size, and income of the country were used as base for sub-analysis. Visual inspection of a funnel plot asymmetry and Egger’s regression test were used to check for potential publication bias [32, 33]. The L estimator of Duval and Tweedie was used to find and fill the missed studies through the procedure called the trim-and-fill analysis [34]. A meta-analysis was conducted after log-transforming the estimates from primary studies. As overall adverse infant health outcomes are considered rare in this studies (most of the are close to one), ORs, RRs, and HRs were assumed as a reasonable approximations of each other [35–37]. Sensitivity analysis was also conducted to test for the presence of studies with outlier estimates. All analyses were conducted in Stata 14 [38].

### Protocol registration

This review was registered in PROSPERO with protocol number CRD42017082624.

## Result

**Fig. 1:**
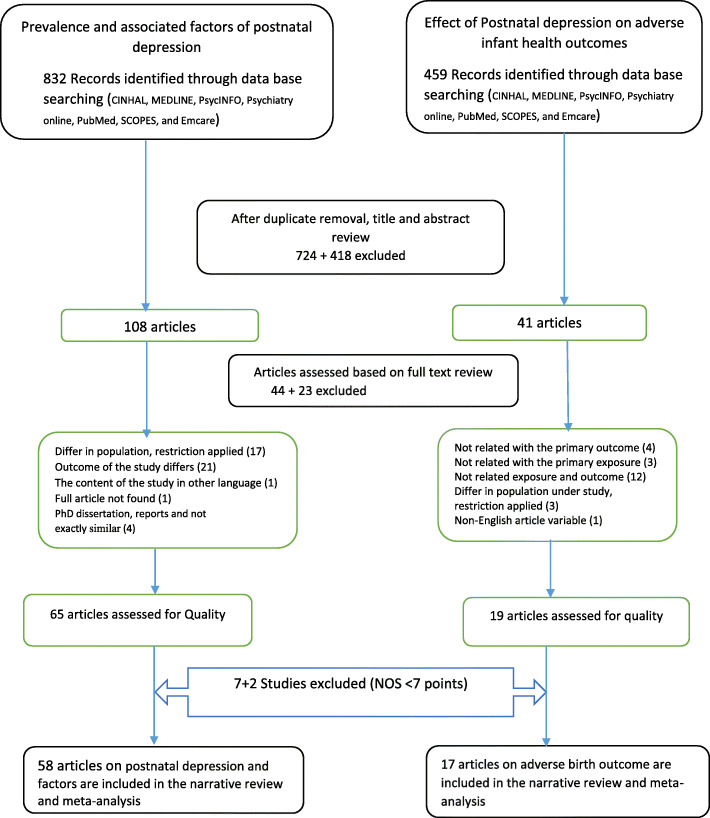
Flow chart of study inclusion for systematic review and meta-analysis of postnatal depression and its effect on adverse infant health outcomes in low and middle-income countries, 2007–2017

In total, 1291 records were retrieved from databases. After removing duplicates, reviewing titles, and abstracts, 149 records were deemed eligible for full text review. After exclusion of 67 records in full text review, 84 records were assessed for quality. Lastly, 58 articles on PND [39–95] and 17 [56, 58, 83, 85, 86, 89–91, 95–103] articles on adverse infant health outcomes were assessed as good quality and were included in quantitative analysis (Fig. 1).

### Postnatal depression

Among 58 studies (with 63,293 population), 47 (81%) studies were conducted in middle-income countries, 38(66%) were institutional-based studies, and 41(70%) used EPDS for screening depression. Fourteen studies were conducted in Africa, 13 studies were from countries located in North and South America, and 31 studies were conducted in Asia. A wide range of PND prevalence (3.5% in Ghana to 58.8% in Iran) was observed across the studies. The studies were published from 2007 to 2017 with sample sizes ranging between 87 and 16,560 participants. (Table 1) Because of substantial evidence of heterogeneity among primary studies as evidenced by Cochran Q (*I*^*2*^ = 99.0%), visual inspection of forest plot, and Higgins test (*p* = 0.001), estimate from DerSimonian and Lairds random-effect model was reported in a sub-group analysis. Because of evidence in publication bias, an estimate from trim and fill analysis was reported in each sub-analysis (Eggers test, *P* < 0.01). A sensitivity analysis showed a PND prevalence ranging between 18.82 and 25.02%.

**Table 1:** Summary of studies conducted on postnatal depression and associated factors in low and middle-income countries (2007–2017), *N* = 58

Author, P. year	Year	Country, income	Study setting	Sample size	Time of assessment	Tool used	Prevalence
Dindar I et al. 2007	2007	Middle	HI	679	birth to 48 weeks	EPDS	25.6%
Ege E et al. 2008	2008	Middle	community	364	6 to 48 weeks	EPDS	33.2%
Flores-Quijano ME et al., 2008	2008	Middle	HI	163	2 to 12 weeks	EPDS	24.5%
Hasselmann MH et al., 2008	2008	Middle	HI	429	birth to 8 weeks	EPDS	35.8%
Tannous L et al., 2008	2008	Middle	community	271	6 to 8 weeks	EPDS	20.7%
Durat and Kutlu, 2010	2009	Middle	HI	126	birth to 48 weeks	EPDS	23.8%
Yagmur Y et al., 2010	2010	Middle	community	730	birth to 48 weeks	EPDS	21.0%
Botino M et al., 2012	2012	Middle	HI	811	birth to 20 weeks	EPDS	24.3%
Goker A et al., 2012	2012	Middle	HI	318	4 to 48 weeks	EPDS	31.4%
Pocan Ag et al., 2013	2013	Middle	HI	187	4 to 6 weeks	EPDS	28.9%
Melo Jr. EF et al., 2012	2012	Middle	HI	555	4 to 6 weeks	EPDS	10.8%
Mathisen SE et al. 2013	2013	Middle	HI	87	4 to 6 weeks	EPDS	37.2%
Serhan N et al. 2013	2012	Middle	community	110	8 to 24 weeks	EPDS	9.1%
de Castro et al 2015	2014	Middle	HI	604	birth to 36 weeks	EPDS	10.6%
Lara MA et al., 2015	2014	Middle	HI	210	24 weeks	SCID-I	13.4%
Corrêa H et al., 2016	2016	Middle	HI	3060	birth to 48 weeks	EPDS	19.5%
Robert A et al., 2016	2016	Middle	HI	194	birth to 6 weeks	EPDS	40.2%
Ho-Yen SD et al. 2007	2007	Low	community	426	4 to five weeks	EPDS	4.9%
Baghianimoghadam et al, 2009	2007	Middle	HI	120	1 to 16 weeks	BDI	58.8%
Gao L et al., 2009	2008	Middle	HI	130	6 to 8 weeks	EPDS	13.8%
Kadir AA, et al., 2009	2009	Middle	HI	293	4 to 6 weeks	EPDS	27.3%
Wan EY et al., 2009	2009	Middle	HI	342	6 to 8 weeks	EPDS	15.5%
Petrosyan D, et al.,2011	2011	Middle	HI	437	3 months	EPDS	14.4%
Ahmed HM et al., 2012	2012	Middle	HI	1000	6 to 8 weeks	EPDS	28.4%
Hegde S et al. 2012	2012	Middle	HI	150	6 to 14 weeks	EPDS	15.5%
Zainal NZ et al. 2012	2012	Middle	HI	411	6 to 8 weeks	MINI	6.8%
Swapan G et al. 2013	2013	Middle	HI	202	6 weeks	PRIME MD	15.8%
Panyayong B et al., 2013	2013	Middle	community	1731	6 to 8 weeks	EPDS	8.4%
Abdollahi F et al., 2014	2014	Middle	HI	1801	8 to 12 weeks	EPDS	4.5%
Deng AW et al., 2014	2014	Middle	community	1823	4 weeks	EPDS	27.4%
El-Hachem C et al. 2014	2014	Middle	HI	228	4 weeks	EPDS	12.0%
Giri RK et al. 2015	2015	Low	HI	346	6 to 10 weeks	EPDS	30.0%
Yusuff ASM et al. 2015	2015	Middle	HI	2072	4 to 24 weeks	EPDS	14.3%
Murray L et al. 2015	2015	Middle	HI	431	4 to 24 weeks	EPDS	18.1%
Shivalli S et al. 2015	2015	Middle	HI	102	4 to 6 weeks	EPDS	31.4%
Abdollahi et al. 2014	2014	Middle	HI	1910	12 weeks	EPDS	19.0%
Safadi RR et al. 2016	2016	Low	HI	315	12 weeks	PHQ-9	25.0%
Iranpour S et al. 2017	2017	Middle	community	360	12 weeks	EPDS	34.8%
Liu S et al. 2017	2017	Middle	community	882	4 weeks	EPDS	6.7%
Ramchandani PG et al. 2009	2008	Middle	community	1035	24 weeks	PDQ	16.4%
Stewart RC et al. 2010	2009	Middle	HI	501	36 weeks	DSM-IV	13.9%
Hassanein I et al. 2014	2014	Low	Hi	290	12 weeks	EPDS	39.0%
Mohammed ES et al. 2014	2014	Low	community	200	56 weeks	EPDS	49.5%
Khalifa DS et al. 2015	2015	Low	HI	300	12 weeks	EPDS	9.2%
Stellenberg E et al. 2015	2016	Middle	community	159	6 to 14 weeks	EPDS	50.3%
Weobong B et al. 2013	2016	Middle	community	13, 360	4 weeks	PHQ-9	3.8%
Shamu S et al., 2008	2016	Low	HI	842	6 weeks	CES-D	21.4%
Azale A et al. 2016	2016	Low	Community	385	24 weeks	PHQ_9	12.1%
Surkan PJ et al. 2009	2009	Middle	HI	595	24 to 48 weeks	CES-D	56.0%
Machado MC et al. 2014	2014	Middle	HI	168	4 t0 12 weeks	EPDS	16.1%
Gausia K et al. 2010	2010	Low	Community	318	6 to 8 weeks	EPDS	20.1%
Upadhyay AK et al. 2016	2016	Middle	Community	1833	20 to 84 weeks	SRQ-20	29.8%
Islam MJ et al. 2017	2016	Low	Community	426	24 weeks	EPDS	35.2%
Saeed Q et al. 2016	2016	Low	Community	325	96 weeks	AKUADS	40.0%
Ndokera R et al. 2008	2008	Middle	community	278	8 to 48 weeks	SRQ-20	9.7%
Guo N et al. 2013 (Ghana)	2013	Middle	HI	654	12 weeks	PHQ_9	8.9%
Guo N et al. 2013(Ivory Coast)	2013	Middle	HI	654	12 weeks	PHQ_9	11.8%
Weobong B et al. 2015	2017	Middle	Community	16,560	4 to 12 weeks	DSM-IV	3.5%

*HI* health institution, *PDQ* Pitt depression questionnaire, *AKUADS* Aga Khan University Anxiety and Depression Scale> = 20, *EPDS* Edinburgh Postnatal Depression Scale, *BDI* Beck depression inventory, *CED* Center for Epidemiological Studies Depression scale, *SCID-I* Structured Clinical Interview for the DSM-IV depression module, *MINI* Mini International Neuropsychiatric Interview, *PHQ-9* patient health questioner, *SRQ-20* Self Reporting Questionnaire

Postnatal depression increased during the last seven years, from 18.2% (95%CI: 12.8–23.5%) in 2010–2012 to 25.6% (95%CI: 19.9–27.2%) in 2016–17. The prevalence was higher in low-income countries (Pooled Prevalence (PP) = 25.8%; 95%CI: 17.9–33.8%) and in health institutional-based studies (PP = 22.1%; 95%CI: 18.8–25.3%). Postnatal depression increases from the earliest weeks of birth (PP = 17.6%; 95%CI; 7.7–27.5%) to the end of second year (PP = 25.2%; 95%CI: 19.9–30.5%). Postnatal depression was the highest in studies used Beck Depression Inventory (BDI), Center for Epidemiological Studies Depression scale (CED), the Structured Clinical Interview for DSM-IV Axis I Disorders (SCID-I), Mini International Neuropsychiatric Interview (MINI) (PP = 28.3%; 95%CI: 16.9–39.8%), and in studies with small sample (PP = 23.3%; 95%CI: 20.2–26.5%) (Table 2).

**Table 2:** Sub-analysis of postnatal depression prevalence in low and middle-income countries (*N* = 58, 2007–2017), (random effect model, result after a trim and fill analysis)

Variable of sub-analysis	Number of studies	Sample size	Pooled prevalence; 95%CI
Year of publication			
2007–2009	15	5752	25.1 (18.1–32.2)
2010–2012	10	4840	18.2 (12.8–23.5)
2013–2015	20	14,000	19.6 (15.8–23.5)
2016–2017 (two years)	13	38,701	25.6 (19.9–27.2)
Income of the country			
Low income	11	4173	25.8 (17.9–33.8)
Middle income	47	59,120	20.7 (18.4–23.1)
Study setting			
Health institution	38	21,717	22.1 (18.8–25.3)
Community based	20	41,576	20.9 (17.9,23.9)
Time of screening			
Birth to four weeks	5	16,487	17.6 (7.8–27.5)
5 weeks to 10 weeks	22	26,599	21.9 (18.0–25.7)
11 weeks to 16 weeks	12	7683	17.9 (14.1–21.8)
17 weeks to 96 weeks	19	12,524	25.2 (19.9–30.5)
A tool used for depression screening			
EPDS	41	25,013	22.6 (19.6,25.7)
PHQ-9 and SRQ-20	7	17,479	14.4 (6.2–22.6)
DSM-IV	2	17,061	8.6 (1.6–18.8)
Other/BDI, CED, SCID-I, MINI/	8	3740	28.3 (16.9–39.8)
Sample size			
< =1091	49	19,143	23.4 (20.2–26.5)
> 1091	9	44,150	14.4 (10.6–18.1)

*EPDS* Edinburgh Postnatal Depression Scale, *BDI* Beck depression inventory, *CED* Center for Epidemiological Studies Depression scale, *SCID-I* structured clinical interview for the DSM-IV depression module, *MINI* Mini International Neuropsychiatric Interview, *PHQ-9* patient health questioner, *SRQ-20* self reporting questionnaire

Poor obstetric history (Pooled Odds Ratio (POR) = 1.98; 95%CI: 1.66–2.36) and social support (POR = 2.44; 95%CI: 1.92–3.09), exposure to history of CMD (POR = 3.30; 95%CI: 1.88–5.80) and violence (POR = 2.61; 95%CI: 2.16–3.15), low economic (POR = 2.05; 95%CI: 1.66–2.54) and educational status (POR = 2.06; 95%CI: 1.56–2.73), and problem with maternal and newborn health (POR = 3.16; 95%CI: 1.96–5.08) were risk factors for PND (Table 3).

**Table 3:** Summary of risk factors significantly associated with postnatal depression (*N* = 58, 2007–2017), (random effect model, result after a trim and fill analysis)

Variable of sub-analysis	Number of studies	Sample size	Pooled Odds Ratio (POR), 95%CI	I^2^, *p*-value
Poor obstetric history (*unplanned pregnancy, GDM, GHP, labor complication, history of emesis, multiparty*)	18	28,766	1.98 (1.66–2.36)	64.5%, p = 0.001
History of CMD (*depression during pregnancy, family psychiatric illness, stressful life event*)	13	10,074	3.30 (1.88–5.80)	99.2%, p = 0.001
Poor social support	12	11,206	2.44 (1.92,3.09)	73.8%, p = 0.001
Low economic status	12	7671	2.05 (1.66–2.54)	96.2%, p = 0.001
Problem with maternal and newborn health	11	5954	3.16 (1.96–5.08)	91.7%, p = 0.001
Exposure to any forms of violence (*physical, emotional, sexual*)	7	5730	2.61 (2.16–3.15)	0%, *p* = 0.867
Low educational status of the mother	7	5549	2.06 (1.56–2.73)	48.2%, *P* = 0.07

*CMD* common mental disorder, *GDM* gestational diabetes mellitus, *GHP* gestational hypertension

### The association between postnatal depression on adverse infant health outcomes

Seventeen studies (33 estimates), with a total of 31,454 participants, were included in this analysis. Nine studies from Africa, eight from Asia, and four from countries in North America were found. Fifteen studies represented middle-income countries, and five studies represented low-income countries. Twelve (57%) studies were longitudinal, 12 (57%) were community-based, and their sample size ranged from 166 to 16,560 participants. Center for Epidemiological Studies Depression scale (CED) and EPDS screening tools were used in 7 (33%) and 5 (23.8%) of the studies, respectively (Table 4).

**Table 4:** Summary of studies conducted on the effect of postnatal depression on infant health outcomes in low and middle-income countries, 2007–2017 (*N* = 14)

Author, P. year	Country, income	Study setting	Study design	Sample size	weeks	Screening tool used	Infant adverse health outcomes	Estimate (RR/OR)	LCI	UCI
Hasselmann MH et al. 2008	Brazil, Middle	HI	cohort	429	4 to 8 weeks	EPDS	Non-EB	1.21	1.02	1.45
Surkan PJ et al. 2008	Brazil, Middle	HI	cross sectional	595	6 to 12 months	CES-D	Short stature	1.8	1.1	2.9
Machado MC et al. 2014	Brazil, Middle	HI	longitudinal	168	1 to 3 months	EPDS	Non-EB	1.61	1.19	2.19
Gausia K et al. 2010	Bangladish, Low	Community	longitudinal	318	6 to 8 weeks	EPDS	Diarrhea	1.74	1.25	3.42
Rahman A et al. 2016	Pakistan, Low	Community	cohort	279	6 months	DSM-IV	Non-EB	1.42	0.98	2.06
Upadhyay AK et al. 2016	India, Middle	Community	longitudinal	1833	5 to 21 months	SRQ-20	Stunting	1.53	1.21	1.92
Islam MJ et al. 2017	Bangladish, Low	Community	cross sectional	426	6 months	EPDS	Non-EB	5	2.27	11.11
Saeed Q et al. 2016	Pakistan, Low	Community	cross sectional	325	2 years	AKUAD	Stunting	3.15	1.91	5.18
							Underweight	3.26	1.99	5.34
Adewuya AO et al. 2007	Nigeria, Middle	HI	case control	242	6 to 12 weeks	SCID-NP	Poor weight	3.41	1.3	8.52
							Poor height	3.28	1.03	10.47
Guo N et al. 2013	Ghana, Middle	HI	Longitudinal	654	3 months	PHQ	Febrile illnesses	1.32	1.01	1.74
Guo N et al. 2013	Côte d’Ivoire Middle						Febrile illnesses	1.57	1.20	2.07
Ashaba Set al 2013	Uganda, Low	HI	Case control	166	1–5 yrs	(M.I.N.I.)	Malnutrition	2.4	1.11	5.18
Weobong B et al. 2017	Ghana, Middle	Community	Longitudinal	16,560	4 to 12 weeks	DSM-IV	Diarrhea	1.8	1.45	2.14
							cough	1.49	1.28	1.7
							Fever	1.8	1.49	2.11
							Vomiting	1.98	1.26	2.71
Madeghe BA et al. 2016	Kenya, Middle	HI	Cross-sectional	200	6 to 14 weeks	EPDS	Non-EB	6.14	2.45	13.36
							Underweight	4.4	1.91	11.93
Wemakor A et al. 2016	Ghana, Middle	HI	Cross-sectional	384	0–59 months	CED-S	Stunting	2.48	1.29	4.77
Ndokera R et al. 2008	Zambia, Middle	community	Cross-sectional	278	2–12 months	CED	serious illness	1.64	0.51	5.24
							diarrhea	1.32	0.71	2.48
							underweight	1.48	0.35	6.22
Maureen M Black et al. 2009	Bangladish, Low	Community	cross sectional	221	6–12 months	CED	Stunting	2.17	1.24	3.81
Benett IM et al. 2015	India, Middle	community	Longitudinal	1930	12 months	CED	Stunting	1.18	1.03	1.35
							Underweight	1.11	0.97	1.26
	Ethiopia, Low			1885			Stunting	0.91	0.81	1.02
							Underweight	1.01	0.89	1.15
	Peru, Low			1946			Stunting	1.06	0.93	1.22
							underweight	0.85	0.6	1.19
	Vietnam Low			1961			Stunting	0.91	0.81	1.02
							Underweight	1.29	1.03	1.62

**Fig. 2:**
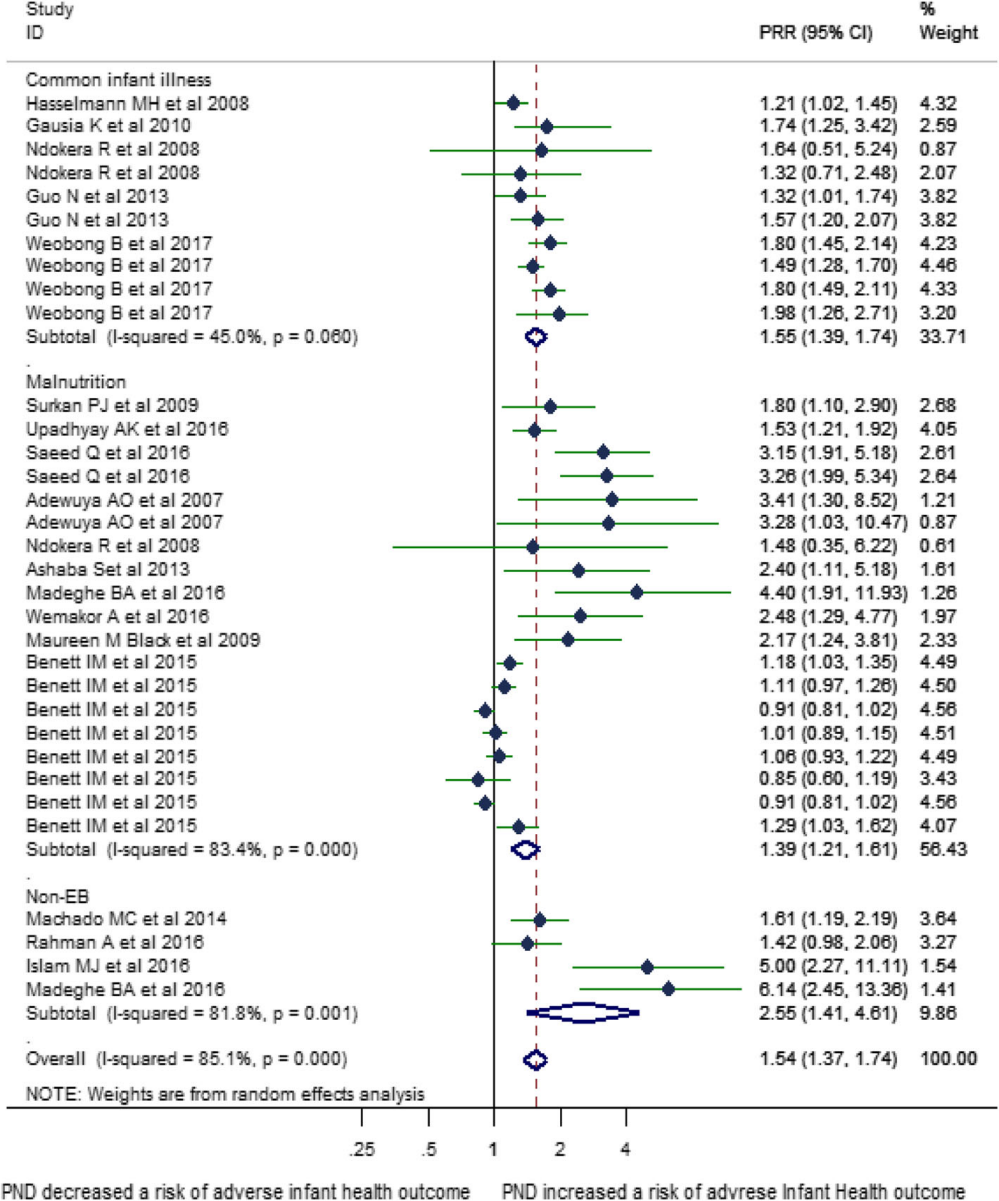
Forest plot of the effect of perinatal depression on adverse infant health outcomes (*N* = 17) in Low and Middle-income countries sub analyzed by the type of adverse infant health outcome

Of 33 estimates, 19 were on malnutrition, 10 were on common infant illness, and 4 were on non-exclusive breastfeeding. The following authors studied more than one outcome in a single study: Adewuya et al. studied two different forms of malnutrition (stunting and underweight); Guo et al. studied febrile illnesses in two countries (Ghana and Côte d’Ivoire); Weobong et al. studied four different types of febrile illnesses (diarrhea, cough, fever, vomiting); Madeghe et al. studied two different forms of outcome (Non-exclusive breast feeding and underweight); Ndokera et al. studied three different forms of outcomes (serios illnesses, diarrhea, underweight); Benett et al. studied two different forms of malnutrition in four countries (stunting and underweight in India, Ethiopia, Peru, and Vietnam) (Table 4) All type of outcomes were sub-analyzed based on the three major forms of adverse infant health outcomes as stated in the Integrated Management of Newborn and Childhood Illnesses (IMNCI) guideline [104]: febrile illnesses (accounts for fever, diarrhea, vomiting, cough); malnutrition (underweight, stunting, malnutrition); and non-exclusive breastfeeding (Fig. 2).

**Fig. 3:**
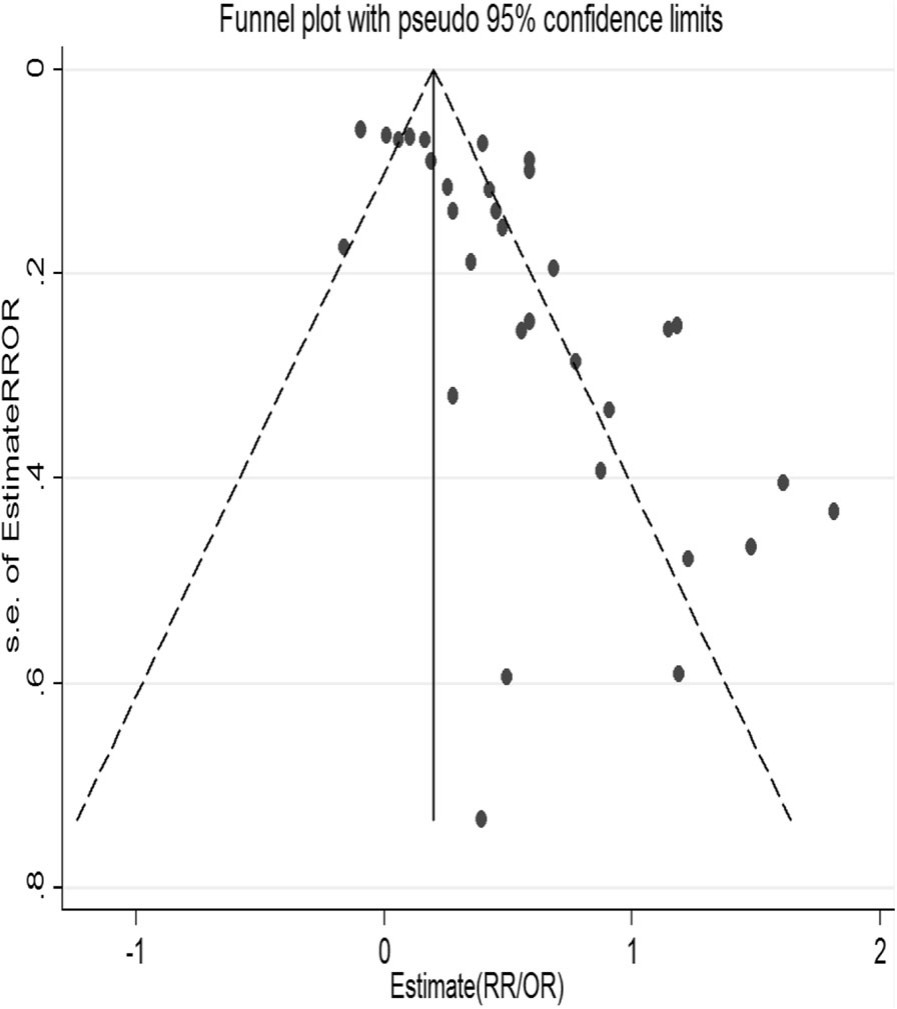
Funnel plot before Tweedie’s and Duval’s trim and fill alanysis for testing publication bias

**Fig. 4:**
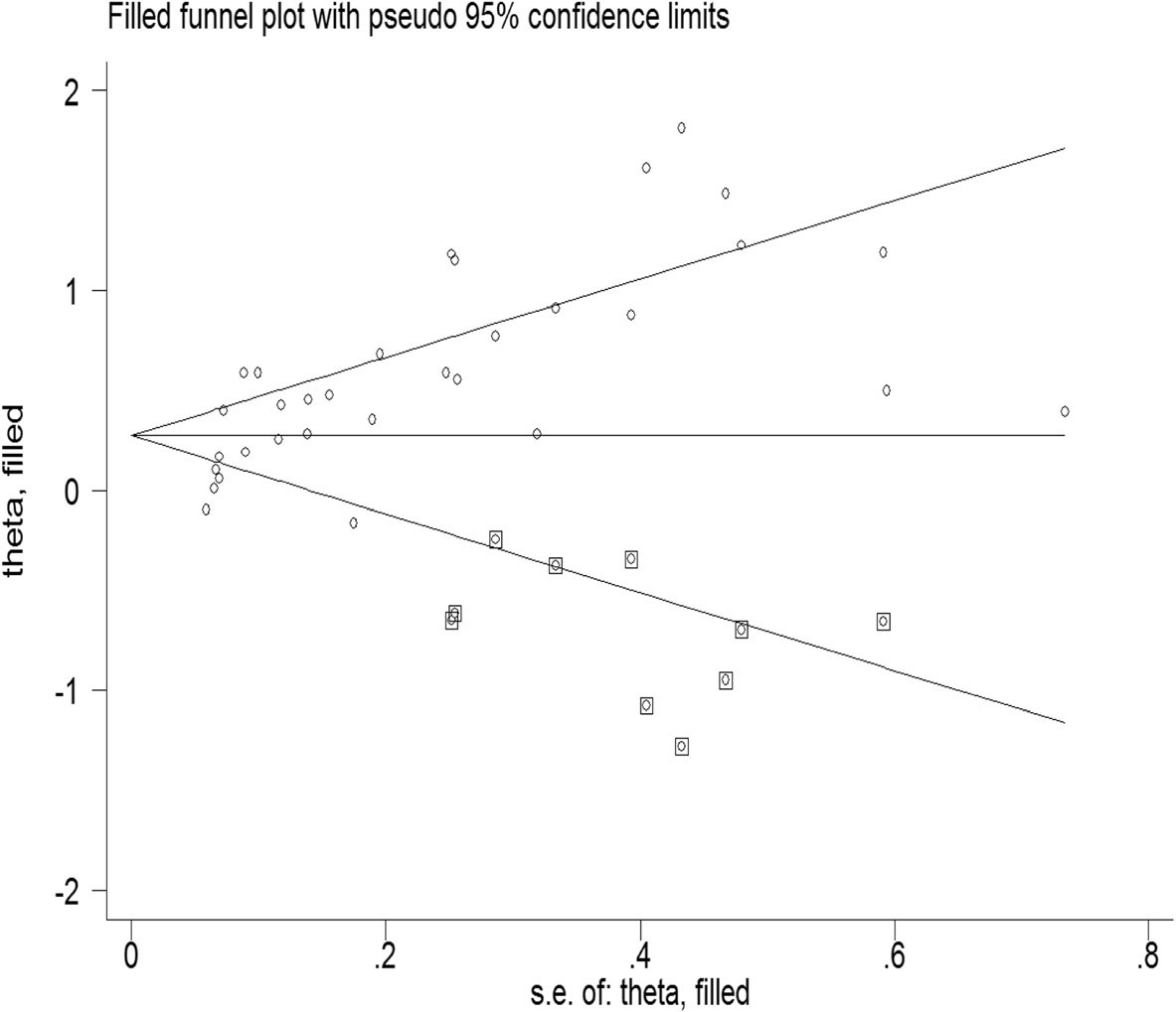
Funnel plot after Tweedie’s and Duval’s trim and fill alanysis (10 unpublished studies found on the topic)

Seventeen estimates were reported in relative risk (RR), 13 in odds ratio (OR), while the rest three estimated the hazard ratio (HR). The association between PND and malnutrition (underweight, wasting, stunting, short stature), common infant illness (diarrhea, febrile illnesses, cough), and non-exclusive breastfeeding were significant in 12, eight, and three studies, respectively. As a small study effect and high heterogeneity were evidenced, the final estimate corrected for trim and fill analyses from a random effect model was reported (Figs. 3 and 4). Accordingly, PND was associated with 1.31 times increased risk of adverse infant health outcomes (95%CI: 1.17–1.48). The sub-analyses based on the types of outcomes showed the following: a risk of being malnourished, being sick by common infant illnesses, and having non-exclusively breastfeeding was 1.39 times (95%CI: 1.21–1.61), 1.55 times (95%CI; 1.39–1.74), and 2.55 times (95%CI; 1.41–4.61) higher among infants of depressed than non-depressed mothers, respectively (Fig. 2).

**Fig. 5:**
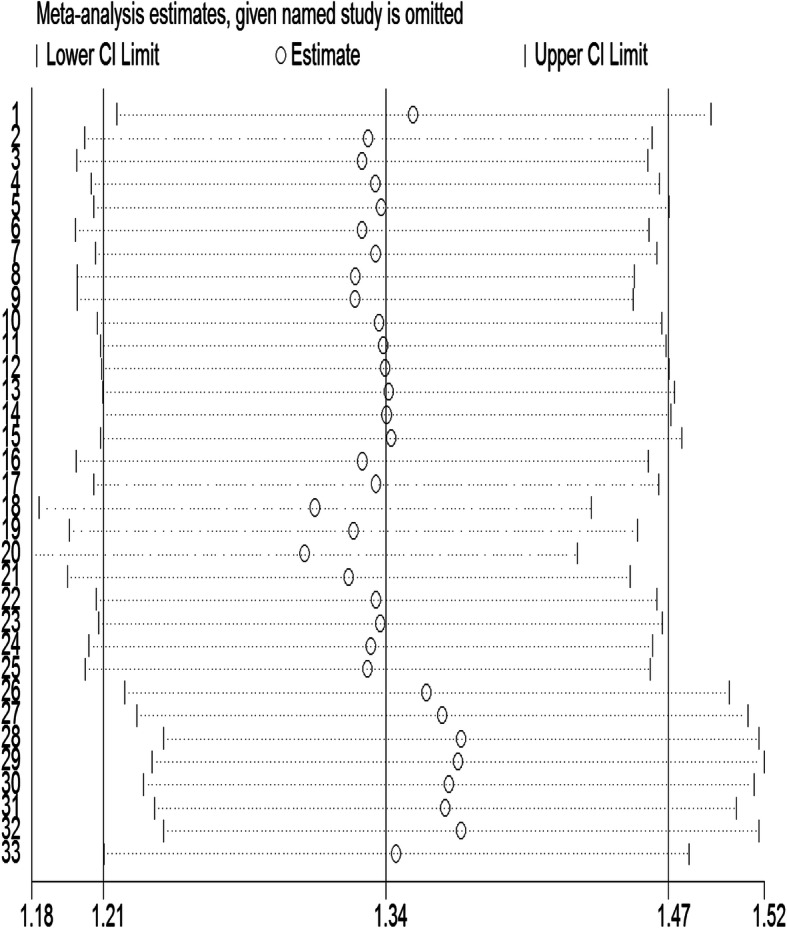
Sensitivity analysis for estimates on postnatal depression and its effect on adverse infant health outcomes in Low-and Middle-income countries (Number of estimates = 33)

A sub-analysis was conducted to explore the consistency of the association across different characteristics of the studies. Accordingly, (1) a pooled estimate from the OR (Pooled Odds Ratio (POR) = 2.62; 95%CI: 2.03–3.38) was larger than the RR (PRR = 1.24; 95%CI: 1.10–1.41) and the HR (PHR = 1.44; 95%CI: 1.21–1.70); (2) the risk was similar for studies used screening (EPDS, PHQ, SRQ) and diagnostic tool (DSM/MINI); (3) the risk of adverse infant health outcomes decreased as age of the infant increased; from 1.75 (95%CI; 1.51–2.03) at the age of 0 to 6 months to 1.28 (95%CI: 1.06–1.54) at the age of 12 months and above; (4) the risk of adverse infant health outcomes was lower in low-income countries (PRR = 1.40; 95%CI:1.37–1.74) compared to middle-income countries (PRR = 1.59; 95%CI: 1.40–1.81); and (5) as sample size increased the association between PND and adverse infant health outcome decreased; from 1.98 (95%CI; 1.63–2.40) for those included a sample size less than 1500 to 1.27 (95%CI; 1.10–1.46) for the studies with larger sample sizes. (Supplementary information) According to the sensitivity analysis, the pooled relative risks ratio was not affected when individual studies were omitted (Fig. 5).

## Discussion

The current comprehensive and relatively sizeable review and meta-analyses have dealt with the burden, risk factors, and effects of PND on infant health outcomes in low and middle-income countries. The review has included 58 primary studies on PND and 17 studies (with 33 estimates) investigating on PND effect on infant health outcomes in the past ten years. The review founds that a significant number of postnatal mothers were depressed, and their infants suffered from malnutrition, common infant illnesses, and non-exclusive breastfeeding.

### Postnatal depression and its predictors

The research evidence dealing with PND in low and middle-income countries has been increasing over time. The current meta-analysis showed that one in four and one in five postnatal mothers were living with PND in low-and middle-income countries, respectively. These findings were consistent with a review conducted by Gelaye et al. in low and middle-income countries [12] and slightly higher than a review published by Sawyer et al. [25]. We hypothesized that the rapid decline in reproductive hormones such as estrogen following childbirth might contribute to a dysregulation of the stress hormone, monoamine, and reproductive hormone, which subsequently leads to depression [105]. Postnatal depression prevalence has been increasing from 18 to 25% in the last seven years, which supports the WHO prediction that depression will be the third global leading cause of morbidity by 2030 [106, 107].

The PND prevalence increased in the first 10 weeks, slightly decreased from 11 to 16 weeks, and steadily rose from 17 to 96 weeks after birth. The trend for the first 16 weeks is similar to a study conducted in developed countries [14, 108]. However, the interpretation for these estimates should account for the window of measurement, as a wider window predicts larger estimates. Community-based studies, studies used diagnostic tools (DSM-IV) for identifying depression, and studies with larger samples predicted relatively prevalence. However, the estimates were consistent across all the sub-analysis that showed the high public health importance of PND in low and middle-income countries.

This review identified the following PND predictors: poor obstetric history and social support, history of common mental disorder and violence, low economic and educational status, and maternal and newborn ill health.

Poor obstetric history (unwanted or unplanned pregnancy, multi-parity, history of emesis) strongly predicted PND. These events could be related to household income, mother’s un-satisfactory birth experiences, predisposition to bad parenting experience, and post-traumatic stress [23, 25]. Unwanted or unplanned pregnancies could also affect emotional and instrumental support that the mother could get from her partner and related families [109]. Psychosocial factors such as a history of common mental health disorders, poor social support, and exposure to violence, were identified as significant predictors of PND. These findings are consistent with previously published systematic reviews [12, 14, 25, 110]. Poor social support could aggravate the mother’s stress and depression symptoms as it affects mother’s self-confidence and efficacy [109, 111]. Pregnant mothers who had depression or a history of mental disorders are more likely to develop PND as they are more likely to dampen their positive affect [112], practice rumination and develop a negative cognitive style that could persist throughout the continuum of pregnancy [113, 114]. Moreover, emerging brain neuroimaging explanations of altered neurocognitive functioning have predicted mother’s exposure to childhood abuse or any type of violence as a risk factor for future psychiatric symptomatology [115, 116].

Low economic status and a problem with maternal and newborn health were also predicted PND. Mothers living in low-income countries are less likely to access adequate housing, health service, nutrition, and they experienced challenges in providing adequate care for their infant situations that would add a further layer of stress [11, 117]. Problem with the mother’s mental health affects mother-infant interactions, hygienic practices during food preparation and storage, and proper care for the newborn, altogether makes the mothers feel guilty and worthless, subsequently leading to depression [91, 118].

### Postnatal depression and its effect on adverse infant health outcomes

Postnatal depression in low-and middle-income countries remains mostly untreated, and there is growing evidence that untreated PND results in adverse infant health outcomes [12, 20–22]. The current systematic review and meta-analysis highlighted the effect of PND on infant health and growth. Postnatal depression increased the risk of adverse infant health outcomes by 31%. PND increased the risk of malnutrition (stunting, wasting, short stature), common infant illnesses, and non-exclusive breastfeeding by 39, 55% and by one and a half fold, respectively. We have estimated that 23.66% (7442) of postnatal mothers who had their infants suffered from adverse infant health outcomes in the study population (31,454) were attributed because of their depression status and could be averted if depression during the postnatal period would have been treated.

PND as a risk of adverse infant health outcomes was consistent across all type of association measurement, between use of screening tools (EPDS, PHQ, and SRQ) and clinically diagnosed depression (DSM and MINI), in both institutional and community-based studies, in both high- and low-income countries, and irrespective of study sample. Besides, the association between PND and adverse infant health outcomes was not changed across the age of the infant, even though the risk of adverse infant health outcomes decreased as the age of the infant increased.

The association between malnutrition [20, 95], infant morbidity [119] and PND was supported by a similar systematic review and meta-analysis [120]. Three pathways have been proposed to explain the link between mother’s PND symptoms and adverse infant health outcomes: (1) genetic, (2) hormonal, neuro-regulatory system impairment, and (3) environmental, an indirect effect of PND on the quality of mother’s caregiving [20, 121]. The endocrine dysregulation because of PND would compromise a psychosocial functioning that affects a mother-infant interaction [122]. In middle and low-income countries, mothers are more responsible for caring, feeding, and nurturing their newborns than fathers [123] though they are suffering from lack of adequate income, access to quality water, poor sanitation, and knowledge of illnesses and their prevention [13]. Under these highlighted conditions and exacerbated depression symptoms, postnatal mothers are unable to provide the expected level of care to their infant that would affect their growth and wellbeing.

The current finding related to the effect of PND on non-exclusive breastfeeding is consistent with two systematic reviews [124, 125]. Postnatal depression affects the self-efficacy of the mother and their intention to breastfeed, as explained in the breastfeeding self-efficacy theory [126, 127]. Mothers of better self-efficacy initiate breastfeeding early, stay breastfeeding for a longer time, and produce self-encouraging thoughts to easily overcome challenges of breastfeeding.

### Strength and limitations

The major strengths of the current review include: (1) it was up-to-date, means included primary studies published up to December 30, 2017; (2) inclusion of 75 studies (22 more studies than the recent systematic review by Gelaye et al. [12]; (3) an in-depth analysis and presentation of PND predictors and; (4) an in-depth and up-to-date estimation and presentation of PND effects on adverse infant health outcomes. However, the estimation would be affected by the type and time of depression measurement, methodological and cultural heterogeneities though we treated this heterogeneity with analytical applications.

Considering the strengths mentioned above and limitations of our systematic review and meta-analysis, we believed in providing the most accurate quantification of PND prevalence, its prominent risk factors and association with infant morbidity, malnutrition, and early breastfeeding cessation in low-and-middle income countries. These findings add one step forward to the consistency of the evidence that PND is a significant public health threat for the birthing mothers and their infants.

## Conclusions

The findings indicate a quarter and one in five postnatal mothers were depressed in low-and middle-income countries, respectively, an indication that it is highly prevalent in the regions. Postnatal mothers with poor obstetric history and social support, history of common mental disorder and childhood violence, low economic status, a problem with maternal and newborn health were more likely to have depression. Postnatal mothers with depression were also at higher risk of having sick, malnourished, and non-exclusively breastfed infant relative to mothers who did not have depression symptoms. More importantly, this effect was similar between studies that clinically diagnosed depression and used self-reporting scales. Based on the current findings, an early screening of postnatal mothers starting from the first four weeks of birth and taking prompt intervention would save the mother and her infant from morbidity, mortality, disability, and future developmental consequences.

## Supplementary information

**Additional file 1 : Supplementary material 1**: Postnatal supplementary information.

## Data Availability

All data generated or analyzed during this review are included in this manuscript and its supplementary information files.

## Abbreviations

CES-D 20: Center for Epidemiologic Studies depression scale 20
DM: Diabetic mellitus
DSM-IV: Diagnostic and statistical manual of mental disorders version 4;
EPDS: Edinburgh postnatal depression scale
HIV: Human immune deficiency)
IPV: Intimate partner violence
LAMICS: Low and middle-income countries
NOS: New Castle Ottawa Scale
OR: Odds ratio
PDQ: Pitt depression questionnaire
PHQ-9: Patient health questioner-9
PND: Postnatal depression
POR: Pooled odds ratio
PRISMA: Preferred reporting items for systematic review and meta-analysis
RCT: Randomized control trial
RR: Relative risk
SRQ-20: Self reporting questioner
WHO: World Health Organization
