# Supplementary material for: Postnatal depression and its association with adverse infant health outcomes in low- and middle-income countries: a systematic review and meta-analysis
Source: BMC Pregnancy Childbirth. 2020 Jul 22;20:416. doi: 10.1186/s12884-020-03092-7 (PMC7374875; doi:10.1186/s12884-020-03092-7)
Supplement: Supplementary file 1 — Additional file 1 : Supplementary material 1: Postnatal supplementary information. [file 12884_2020_3092_MOESM1_ESM.docx]

Postnatal Depression and its Effect on Infant Health Outcomes: A Systematic Review and Meta-analysis of Studies from Low and Middle Income Countries

Abel Fekadu Dadi^12^* Emma R Miller^1^ Lillian Mwanri^1^

^1^Flinders University, Health Sciences Building, Sturt Road, Bedford Park, Adelaide, SA, 5001

^2^Institute of Public Health, College of Medicine and Health Sciences, University of Gondar, Gondar, Ethiopia

**Supplementary information**

Table of Contents

[Table 1: PRISMA checklist 3](#_Toc514678294)

[Letrature searching strategy for postnatal depression and its associated factors 6](#_Toc514678295)

[Letrature searching strategy for postnatal depression and its effect on adverse infant health outcomes 8](#_Toc514678296)

[Excluded studies and reason for exclusion 10](#_Toc514678297)

[NOS quality assessment checklist 11](#_Toc514678298)

[1. Postnatal depression 11](#_Toc514678299)

[Table 10: Summary of studies conducted on postnatal depression and associated factors in Low and Middle income countries (2007-2017), N= 58 18](#_Toc514678300)

[Fig 1: Meta-analysis of postnatal depression prevalence (Forest plot) 21](#_Toc514678301)

[Fig 2: Galbraith plot for testing heterogeneity among postnatal depression estimates 22](#_Toc514678302)

[Testing publication bias for postnatal depression prevalence 23](#_Toc514678303)

[Egger's test for small-study effects: 23](#_Toc514678304)

[Fig 3: Funnel plot 23](#_Toc514678305)

[Fig 3: Sensitivity analysis for postnatal depression prevalence estimates 24](#_Toc514678306)

[Risk factors for postnatal depression 25](#_Toc514678307)

[Fig 4: Bad obstetric history as a factor for postnatal depression 25](#_Toc514678308)

[Fig 5: Poor social support as a risk factor for postnatal depression 26](#_Toc514678309)

[Fig 6: History of common mental disorder as a risk factor for postnatal depression 27](#_Toc514678310)

[Fig 7: Problem with maternal and new born health as a risk factor for postnatal depression 28](#_Toc514678311)

[Fig 8: Exposure to any form of violence as a risk factor for postnatal depression 29](#_Toc514678312)

[Fig 9: Low economic status as a risk factor for postnatal depression 30](#_Toc514678313)

[Fig 10: Low educational status as a risk factor for postnatal depression 31](#_Toc514678314)

[Chapter II: Postnatal depression and adverse infant health outcome 32](#_Toc514678315)

[Table 2.1: Summary of studies conducted on the effect of postnatal depression on adverse infant health outcomes 32](#_Toc514678316)

[Table 2.2: Trim and fill analysis for postnatal depression effect on adverse infant health outcome 35](#_Toc514678317)

[Fig 2.1: Forest plot after trim and fill analysis (showed 10 studies left unpublished) 36](#_Toc514678318)

[Fig 2.2: Galbraith Plot for testing heterogeneity among studies on effect of postnatal depression on adverse infant health outcomes 37](#_Toc514678319)

[Fig 2.3: Sensitivity analysis of studies on effect of postnatal depression on adverse infant health outcomes 38](#_Toc514678320)

[Sub Analysis of effect of postnatal depression on adverse infant health outcomes 39](#_Toc514678321)

# Table 1: PRISMA checklist

| **Section/topic** | **#** | 1. **Checklist item** | **Reported on page #** |
| --- | --- | --- | --- |
| 1. **TITLE** | | |  |
| Title | 1 | 1. Identify the report as a systematic review, meta-analysis, or both. | 1(Title) |
| 1. **ABSTRACT** | | |  |
| Structured summary | 2 | 1. Provide a structured summary including, as applicable: background; objectives; data sources; study eligibility criteria, participants, and interventions; study appraisal and synthesis methods; results; limitations; conclusions and implications of key findings; systematic review registration number. | 2 |
| 1. **INTRODUCTION** | | |  |
| Rationale | 3 | 1. Describe the rationale for the review in the context of what is already known. | 4(Paragraph 2) |
| Objectives | 4 | 1. Provide an explicit statement of questions being addressed with reference to participants, interventions, comparisons, outcomes, and study design (PICOS). | 4(Paragraph 2) |
| 1. **METHODS** | | |  |
| Protocol and registration | 5 | 1. Indicate if a review protocol exists, if and where it can be accessed (e.g., Web address), and, if available, provide registration information including registration number. | 6(paragraph 4) |
| Eligibility criteria | 6 | 1. Specify study characteristics (e.g., PICOS, length of follow-up) and report characteristics (e.g., years considered, language, publication status) used as criteria for eligibility, giving rationale. | 5(Paragraph 1) |
| Information sources | 7 | 1. Describe all information sources (e.g., databases with dates of coverage, contact with study authors to identify additional studies) in the search and date last searched. | 4(Paragraph 3) |
| Search | 8 | 1. Present full electronic search strategy for at least one database, including any limits used, such that it could be repeated. | 4(Paragraph 3)  Appendix p 6-8 |
| Study selection | 9 | 1. State the process for selecting studies (i.e., screening, eligibility, included in systematic review, and, if applicable, included in the meta-analysis). | 5(Paragraph 1) & (Figure 1)  Appendix 10 &11 |
| Data collection process | 10 | 1. Describe method of data extraction from reports (e.g., piloted forms, independently, in duplicate) and any processes for obtaining and confirming data from investigators. | 5(paragraph 3)  Appendix 18 & 32 |
| Data items | 11 | 1. List and define all variables for which data were sought (e.g., PICOS, funding sources) and any assumptions and simplifications made. | 5(paragraph 3) Appendix 6 -8 |
| Risk of bias in individual studies | 12 | 1. Describe methods used for assessing risk of bias of individual studies (including specification of whether this was done at the study or outcome level), and how this information is to be used in any data synthesis. | 6(Paragraph 3)  Appendix 10 - 16 |
| Summary measures | 13 | 1. State the principal summary measures (e.g., risk ratio, difference in means). | 5&6(paragraph 3 and 4) |
| Synthesis of results | 14 | 1. Describe the methods of handling data and combining results of studies, if done, including measures of consistency (e.g., I^2^) for each meta-analysis. | 6(paragraph 2 &3) |

| **Section/topic** | **#** | **Checklist item** | **Reported on page #** |
| --- | --- | --- | --- |
| Risk of bias across studies | 15 | Specify any assessment of risk of bias that may affect the cumulative evidence (e.g., publication bias, selective reporting within studies). | 6(Paragraph 3) |
| Additional analyses | 16 | Describe methods of additional analyses (e.g., sensitivity or subgroup analyses, meta-regression), if done, indicating which were pre-specified. | 6(Paragraph 3) |
| **RESULTS** | | |  |
| Study selection | 17 | Give numbers of studies screened, assessed for eligibility, and included in the review, with reasons for exclusions at each stage, ideally with a flow diagram. | 7&13  Figure 1 |
| Study characteristics | 18 | For each study, present characteristics for which data were extracted (e.g., study size, PICOS, follow-up period) and provide the citations. | 9 and 15 |
| Risk of bias within studies | 19 | Present data on risk of bias of each study and, if available, any outcome level assessment (see item 12). | 6  Appendix  10 - 16 |
| Results of individual studies | 20 | For all outcomes considered (benefits or harms), present, for each study: (a) simple summary data for each intervention group (b) effect estimates and confidence intervals, ideally with a forest plot. | 13-14  Appendix p21; 25-31 & 39, |
| Synthesis of results | 21 | Present results of each meta-analysis done, including confidence intervals and measures of consistency. | 13-14  Appendix p21; 25-31 & 39, |
| Risk of bias across studies | 22 | Present results of any assessment of risk of bias across studies (see Item 15). | 6  Appendix  10 - 16 |
| Additional analysis | 23 | Give results of additional analyses, if done (e.g., sensitivity or subgroup analyses, meta-regression [see Item 16]). | 6  Appendix  24 & 38, 25-31 & 39, |
| **DISCUSSION** | | |  |
| Summary of evidence | 24 | Summarize the main findings including the strength of evidence for each main outcome; consider their relevance to key groups (e.g., healthcare providers, users, and policy makers). | 17(Paragraph 1) |
| Limitations | 25 | Discuss limitations at study and outcome level (e.g., risk of bias), and at review-level (e.g., incomplete retrieval of identified research, reporting bias). | 20(Paragraph 3&4) |
| Conclusions | 26 | Provide a general interpretation of the results in the context of other evidence, and implications for future research. | 21(Paragraph 1) |
| **FUNDING** | | |  |
| Funding | 27 | Describe sources of funding for the systematic review and other support (e.g., supply of data); role of funders for the systematic review. | 22(Paragraph 4) |

# **Leterature search strategy**

We searched CINHAL, MEDLINE, Emcare, [PubMed](https://www.ncbi.nlm.nih.gov/pubmed?otool=iauflullib&myncbishare=flinlib), Psych Info [and Scopus](http://ezproxy.flinders.edu.au/login?url=https://www.scopus.com/scopus/home.url) data bases using the following search terms: Postnatal depression, postpartum depressive symptom, exclusive breast feeding, pneumonia, common infant illnesses, diarrhea, measles, cough, fever, malnutrition, and infant feeding practices restricted to studies conducted in Low and Middle income countries, published in between January 2007 and December 2017, published in English, and observational studies. Search strategy for data bases are given in table 1.

Table 1: Search strategy (example for one data base)

| **#** | **Data base** | **Search builder** |
| --- | --- | --- |
|  | 1. **Search strategy for postnatal depression** | |
| 1 | [PsycINFO](http://ezproxy.flinders.edu.au/login?url=http://ovidsp.ovid.com/ovidweb.cgi?T=JS&MODE=ovid&D=psyh&PAGE=main&NEWS=Y) | (exp POSTPARTUM DEPRESSION/) or (Depress*.tw,id.) AND (postnat* or postnatal wom?n or postpartum wom?n).tw,id.) AND ((exp Risk Factors/) or (risk*.tw,id.)) AND ((cross sectional* or case control* or nested-case contorl).mp.) : all Sort by: PublicationDate Filters: Publication date from 2007/01/01 to 2017/12/31; Humans; English; Female; Field: Title/Abstract |
| 2 | [Scopus](http://ezproxy.flinders.edu.au/login?url=https://www.scopus.com/scopus/home.url) | ((Postnatal mothers) OR (Postpartum mothers) OR (mothers after birth)) AND ((Depression during postnatal period) OR (postnatal depression ) OR (depression after birth ) OR (postpartum depressive symptom) OR (depressive mood following birth)) AND ((risk factors) OR correlates OR (associated factors) OR predictors)) AND ((cross sectional*) OR survey OR (case control*) OR (nested case control*) OR (prospective follow up) OR ( follow up) OR (retrospective follow up)) : all Sort by: PublicationDate Filters: Publication date from 2007/01/01 to 2017/12/31; Humans; English; Female;Field: Title/Abstract |
| 3 | **Emcare** | (exp POSTPARTUM DEPRESSION/) or (Depress*.tw,id.) AND (postnat* or postnatal wom?n or postpartum wom?n).tw,id.) AND ((exp Risk Factors/) or (risk*.tw,id.)) AND ((cross sectional* or case control* or nested-case contorl).mp.) : all Sort by: PublicationDate Filters: Publication date from 2007/01/01 to 2017/12/31; Humans; English; Female;Field: Title/Abstract |
|  | **2. Search strategy for the effect of postnatal depression on infant health outcome** | |
| 1 | [MEDLINE](http://ezproxy.flinders.edu.au/login?url=http://ezproxy.flinders.edu.au/login?url=http://ovidsp.ovid.com/ovidweb.cgi?T=JS&PAGE=main&D=ppezv&MODE=ovid&NEWS=N) | (exp POSTPARTUM DEPRESSION/) or (Depress*.tw,id.) AND (postnat* or postnatal wom?n or postpartum wom?n).tw,id.) AND (*Neonatal Intensive Care/ or *Birth Weight/ or exp Infant Development/ or exp Morbidity/ or *Premature Birth/ or exp Neonatal Development/ or exp Neonatal Disorders/) or ((Common post neonatal illness or neonatal illnes* or malaria or pneumonia or fever or diarrhea or measle).mp. [mp=title, abstract, original title, name of substance word, subject heading word, keyword heading word, protocol supplementary concept word, rare disease supplementary concept word, unique identifier, synonyms]) AND ((exp Psychosocial Factors/ or exp Risk Factors/) or (risk*.tw,id.)) AND ((prospective cohort* or retrospective cohort* follow up* or longitudinal* or cross sectional* or case control* or nested-case control).mp.) : all Sort by: PublicationDate Filters:Publication date from 2007/01/01 to 2017/12/31; Humans; English; Female;Field: Title/Abstract |
| 2 | [PsycINFO](http://ezproxy.flinders.edu.au/login?url=http://ovidsp.ovid.com/ovidweb.cgi?T=JS&MODE=ovid&D=psyh&PAGE=main&NEWS=Y) | (exp POSTPARTUM DEPRESSION/) or (Depress*.tw,id.) AND (postnat* or postnatal wom?n or postpartum wom?n).tw,id.) AND (*Neonatal Intensive Care/ or *Birth Weight/ or exp Infant Development/ or exp Morbidity/ or *Premature Birth/ or exp Neonatal Development/ or exp Neonatal Disorders/) or ((Common post neonatal illness or neonatal illnes* or malaria or pneumonia or fever or diarrhea or measle).mp. [mp=title, abstract, original title, name of substance word, subject heading word, keyword heading word, protocol supplementary concept word, rare disease supplementary concept word, unique identifier, synonyms]) AND ((exp Psychosocial Factors/ or exp Risk Factors/) or (risk*.tw,id.)) AND ((prospective cohort* or retrospective cohort* follow up* or longitudinal* or cross sectional* or case control* or nested-case control).mp.) : all Sort by: PublicationDate Filters: Publication date from 2007/01/01 to 2017/12/31; Humans; English; Female;  Field: Title/Abstract |

# **Excluded studies and reason for exclusion**

1. Postnatal depression and risk factors
2. Exclusively conducted in restricted population like: adolescent, HIV-cases , immigrant, new mothers, high risk population, excluded population (1-17)
3. Not specific to postnatal depression (conducted on quality of life, distress, stress, tool evaluation)(13, 18-38)
4. Non-English language (39)
5. Full content not found (40)
6. PhD dissertation, reports and not exactly similar with the current review (41-44)
7. Poor quality on NOS (studies those had <=7 points on NOS quality scale) (45-51)
8. Adverse infant health outcome
9. Not related to the primary outcome of the review (52-56)
10. Not related to the primary exposure of the review(57-60)
11. Not related to the exposure and outcome of the review(61-69)
12. Exclusively conducted in restricted population (70-73)
13. Non-English article(74)
14. Poor quality on NOS (studies those had <=7 points on NOS quality scale) (75)

# **NOS quality assessment checklist**

## **Postnatal depression**

Table 4: Quality ratings for cross –sectional studies included on the basis of Newcastle-Ottawa quality assessment scale

|  | **Selection**  (max-5points) | | | | **Comparability**  (max-two points) | **Assessment of the outcome**  (max-three points) | | **Total Score** |
| --- | --- | --- | --- | --- | --- | --- | --- | --- |
| List of studies | representativeness of the sample | Sample size | Non-respondents | Ascertainment of the risk-factors | The subjects in different outcome groups are comparable, based on the study design or analysis. Confounding factors are controlled. | Ascertainment of the outcome | Stastical test |  |
| Ahmed, H. M. , 2012 | 1 | 1 | 1 | 1 | 1 | 1 | 1 | 8 |
| Azale, Telake, 2016 | 1 | 1 | 1 | 1 | 2 | 1 | 1 | 8 |
| Baghianimoghadam, M. H., 2007 | 0 | 1 | 1 | 1 | 2 | 1 | 1 | 7 |
| Bottino, M. N., 2012 | 1 | 1 | 1 | 1 | 2 | 1 | 1 | 8 |
| Correa, H., 2016 | 1 | 1 | 1 | 1 | 2 | 1 | 1 | 8 |
| de Castro, F., 2015 | 1 | 0 | 1 | 1 | 1 | 2 | 1 | 7 |
| Deng, Ai-Wen, 2014 | 1 | 0 | 1 | 1 | 2 | 1 | 1 | 7 |
| Dindar, Ilknur, 2007 | 1 | 1 | 1 | 1 | 2 | 1 | 1 | 8 |
| Dubey, C., 2012 | 1 | 0 | 1 | 1 | 1 | 2 | 1 | 7 |
| Ege, E., 2008 | 1 | 1 | 1 | 1 | 2 | 1 | 1 | 8 |
| El-Hachem, C., 2014 | 1 | 1 | 1 | 1 | 1 | 2 | 1 | 8 |
| Flores-Quijano, M. E., 2008 | 0 | 1 | 1 | 1 | 2 | 1 | 1 | 7 |
| Galeshi, M., 2016 | 0 | 1 | 1 | 1 | 1 | 1 | 1 | 6 |
| Gao, Ling-ling, 2008 | 1 | 1 | 1 | 1 | 1 | 1 | 1 | 7 |
| Giri, R. K., 2015 | 1 | 1 | 1 | 1 | 1 | 1 | 1 | 8 |
| Goker, Asli, 2012 | 1 | 0 | 1 | 1 | 1 | 1 | 1 | 7 |
| Gomez-Beloz, A., 2009 | 1 | 1 | 1 | 1 | 1 | 1 | 1 | 8 |
| Gupta, Swapan, 2013 | 1 | 1 | ? | 1 | 1 | 1 | 1 | 8 |
| Hanlon, Charlotte, 2010 | 1 | 1 | 1 | 1 | 1 | 1 | 1 | 8 |
| Hassanein, Ibrahim M. A., 2014 | 1 | 1 | 1 | 1 | 1 | 1 | 1 | 8 |
| Hasselmann, M. H., 2008 | 1 | 0 | 1 | 1 | 1 | 1 | 1 | 7 |
| Supriya Hegde1, 2012 | 0 | 1 | 1 | 1 | 1 | 1 | 1 | 7 |
| Ho-Yen, S. D., 2007 | 1 | 0 | 1 | 1 | 1 | 1 | 1 | 7 |
| Iranpour, S., 2016 | 1 | 0 | 1 | 1 | 1 | 1 | 1 | 7 |
| Iranpour, S., 2017 | 1 | 0 | 1 | 1 | 1 | 1 | 1 | 7 |
| Johnson, A. R., 2015 | 0 | Consecutive sampling | 1 | 1 | 1 | 1 | 1 | 6 |
| Kadir, Azidah Abdul, 2009 | 0 | 0 | 1 | 1 | 2 | 1 | 1 | 7 |
| Kakyo, Tracy Alexis, 2012 | 1 | 1 | 0 | 1 | 1 | 1 | 1 | 6 |
| Kumwar, D., 2015 | 0 | 1 | 1 | 1 | 1 | 1 | 1 | 6 |
| Lara, M. A., 2015 | 1 | 1 | 1 | 1 | 1 | 1 | 1 | 7 |
| Liu, S, 2017 | 1 | 1 | 1 | 1 | 2 | 1 | 1 | 8 |
| Mathisen, S. E., 2013 | 0 | 1 | 1 | 1 | 2 | 1 | 1 | 6 |
| Melo Jr, E. F., 2012 | 1 | 1 | 1 | 1 | 2 | 1 | 1 | 8 |
| Mohammed, E. S., 2014 | 1 | 1 | 1 | 1 | 2 | 1 | 1 | 8 |
| Muneer, A., 2009 | 0 | 1 | 1 | 1 | 2 | 1 | 1 | 7 |
| Murray, L., 2015 | 1 | 1 | 1 | 1 | 2 | 1 | 1 | 8 |
| Panyayong, B., 2013 | 1 | 1 | 1 | 1 | 2 | 1 | 1 | 8 |
| Safadi, R. R., 2016 | 1 | 1 | 1 | 1 | 1 | 1 | 1 | 7 |
| Serhan, Nilüfer, 2013 | 1 | 1 | 0 | 1 | 1 | 2 | 1 | 7 |
| Shamu, Simukai, 2016 | 1 | 1 | 1 | 1 | 1 | 1 | 1 | 7 |
| Shivalli, Siddharudha, 2015 | 0 | 1 | 1 | 1 | 1 | 2 | 1 | 7 |
| Stellenberg, E. L., 2015 | 1 | 0 | 1 | 1 | 2 | 1 | 1 | 7 |
| Stewart, Robert C., 2010 | 0 | 0 | 1 | 1 | 2 | 2 | 1 | 7 |
| Tannous, Leila, 2008 | 1 | 1 | 1 | 1 | 1 | 2 | 1 | 8 |
| Wan, E. Y., 2009 | 1 | 0 | 1 | 1 | 1 | 1 | 1 | 7 |
| Yagmur, Y., 2010 | 1 | 1 | 1 | 1 | 1 | 2 | 1 | 8 |
| Zainal, Nor Zuraida, 2012 | 1 | 0 | 1 | 1 | 2 | 1 | 1 | 7 |

Table 5: Quality ratings for case control studies included on the basis of Newcastle-Ottawa quality assessment scale

|  | **Selection**(score) | | | | **Comparability**  (score) | **Exposure**  (score) | | | **Total Score/out of 8** |
| --- | --- | --- | --- | --- | --- | --- | --- | --- | --- |
| List of studies | Case definition | Representative of cases | Selections of controls | Definition of controls | Control any confounding variable | Ascertainment of exposure | Same method of ascertainment for participants | Nonresponse rate (not higher than 25%) – justifiable power maintained |  |
| Petrosyan, Diana, 2011 | 1 | 1 | 0 | 1 | 1 | 1 | 1 | 1 | 7 |
| Roomruangwong, C., 2016 | 1 | 1 | 0 | 1 | 1 | 1 | 1 | 1 | 6 |
| Suhitharan, Thangavelautham, 2016 | 1 | 1 | 0 | 1 | 1 | 1 | 1 | 0 | 6 |

Table 6: Quality ratings for the cohort studies included on the basis of Newcastle-Ottawa quality assessment scale

|  | **Selection**(score) | | | | **Comparability**  (score) | **Outcome**(score) | | | **Total Score (out of 8)** |
| --- | --- | --- | --- | --- | --- | --- | --- | --- | --- |
| List of studies | Representative of exposed cohort | Selections of non-exposed cohort | Assessment of exposure | Absence of outcome at start of study | Control for any confounding variable | Assessment of outcome | Follow-up period (at least three months after birth) | Adequacy of  follow-up ( to clearly get the outcome of interest) |  |
| Abdollahi et al 2016 | 1 | 1 | 1 | **1** | 1 | 1 | 1 | 1 | 8 |
| Abdollahi F et al, 2014 | 1 | 1 | 1 | 1 | 1 | 1 | 1 | 1 | 8 |
| Khalifa DS et al 2015 | 1 | 1 | 1 | 1 | 1 | 1 | 1 | 0 | 7 |
| Mohamad Yusuff, Aza Sherin, 2015 | 1 | 1 | 1 | 1 | 1 | 1 | 1 | 1 | 8 |
| Ramchandani, Paul G. , 2009 | 1 | 1 | 1 | 1 | 1 | 0 | 1 | 1 | 7 |
| Weobong B et al 2015 | 1 | 1 | 1 | 1 | 1 | 1 | 1 | 0 | 7 |

1. **Adverse infant health outcomes**

Table 7: Quality ratings for cross –sectional studies included on the basis of Newcastle-Ottawa quality assessment scale

|  | **Selection**  (max-5points) | | | | **Comparability**  (max-two points) | **Assessment of the outcome**  (max-three points) | | **Total Score** |
| --- | --- | --- | --- | --- | --- | --- | --- | --- |
| List of studies | representativeness of the sample | Sample size | Non-respondents | Ascertainment of the risk-factors | The subjects in different outcome groups are comparable, based on the study design or analysis. Confounding factors are controlled. | Ascertainment of the outcome | Stastical test |  |
| Flores-Quijano, M. E., 2008 | 0 | 1 | 1 | 1 | 1 | 1 | 1 | 6 |
| Islam MJ et al 2016 | 1 | 1 | 1 | 1 | 2 | 1 | 1 | 8 |
| Madeghe BA et al 2016 | 1 | 1 | 1 | 1 | 1 | 2 | 1 | 8 |
| Ndokera R et al 2008 | 1 | 1 | 1 | 1 | 1 | 1 | 1 | 7 |
| Surkan PJ et al 2009 | 1 | 1 | 1 | 1 | 1 | 2 | 1 | 8 |
| Saeed Q et al 2016 | 1 | 1 | 1 | 1 | 2 | 1 | 1 | 8 |
| Wemakor A et al 2016 | 1 | 1 | 1 | 1 | 2 | 1 | 1 | 8 |

Table 8: Quality ratings for case control studies included on the basis of Newcastle-Ottawa quality assessment scale

|  | **Selection**  (score) |  |  |  | **Comparability**  (score) | **Exposure**  (score) |  |  | **Total Score/out of 8** |
| --- | --- | --- | --- | --- | --- | --- | --- | --- | --- |
| List of studies | Case definition | Representative of cases | Selections of controls | Definition of controls | Control any confounding variable | Ascertainment of exposure | Same method of ascertainment for participants | Non-response rate (not higher than 25%) – justifiable power maintained |  |
| Abiodun O. Adewuya, et al 2007 | 1 | 1 | 0 | 1 | 1 | 1 | 1 | 1 | 7 |
| Ashaba, S, 2015 | 1 | 1 | 0 | 1 | 1 | 1 | 1 | 1 | 7 |

Table 9: Quality ratings for the cohort studies included on the basis of Newcastle-Ottawa quality assessment scale

|  | **Selection**(score) | | | | **Comparability**  (score) | **Outcome**(score) | | | **Total Score (out of 8)** |
| --- | --- | --- | --- | --- | --- | --- | --- | --- | --- |
| List of studies | Representative of exposed cohort | Selections of non-exposed cohort | Assessment of exposure | Absence of outcome at start of study | Control for any confounding variable | Assessment of outcome | Follow-up period (at least three months after birth) | Adequacy of  follow-up ( to clearly get the outcome of interest) |  |
| Gausia, K. 1010 | 0 | 1 | 1 | **1** | 1 | 1 | 1 | 1 | 7 |
| Guo N et al 2013 | 1 | 1 | 1 | 1 | 1 | 1 | 1 | 0 | 7 |
| Hasselmann MH et al 2008 | 1 | 1 | 1 | 1 | 1 | 1 | 1 | 0 | 7 |
| Machado MC et al 2014 | 1 | 1 | 1 | 1 | 1 | 1 | 1 | 0 | 7 |
| Rahman A et al 2016 | 1 | 1 | 1 | 1 | 1 | 1 | 1 | 1 | 8 |
| Upadhyay AK et al 2016 | 1 | 1 | 1 | 1 | 1 | 1 | 1 | 1 | 8 |
| Weobong B et al 2017 | 1 | 1 | 1 | 1 | 1 | 1 | 1 | 1 | 8 |
| Benett IM et al 2015 | 1 | 1 | 1 | 1 | 1 | 1 | 1 | 1 | 8 |
| Maureen M Black et al 2009 | 1 | 1 | 1 | 1 | 1 | 1 | 1 | 1 | 8 |


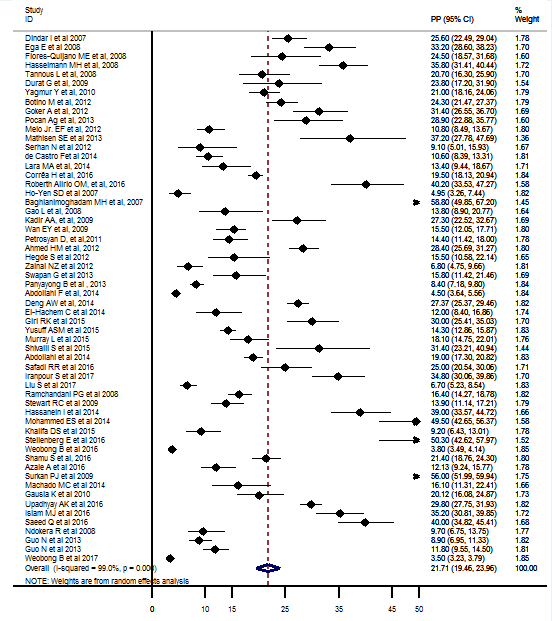


PPD= pooled postnatal depression

# Fig 1: Meta-analysis of postnatal depression prevalence (Forest plot)

# **Testing publication bias for postnatal depression prevalence**

## Egger's test for small-study effects:

Number of studies = 58 Root MSE = 588.3

Std_Eff Coef. Std. Err. t P>t [95% Conf. Interval]

slope 2.527562 .8539526 2.96 0.005 .8168894 4.238235

bias 973.746 98.58112 9.88 0.000 776.2643 1171.228

Test of H0: no small-study effects P = 0.000


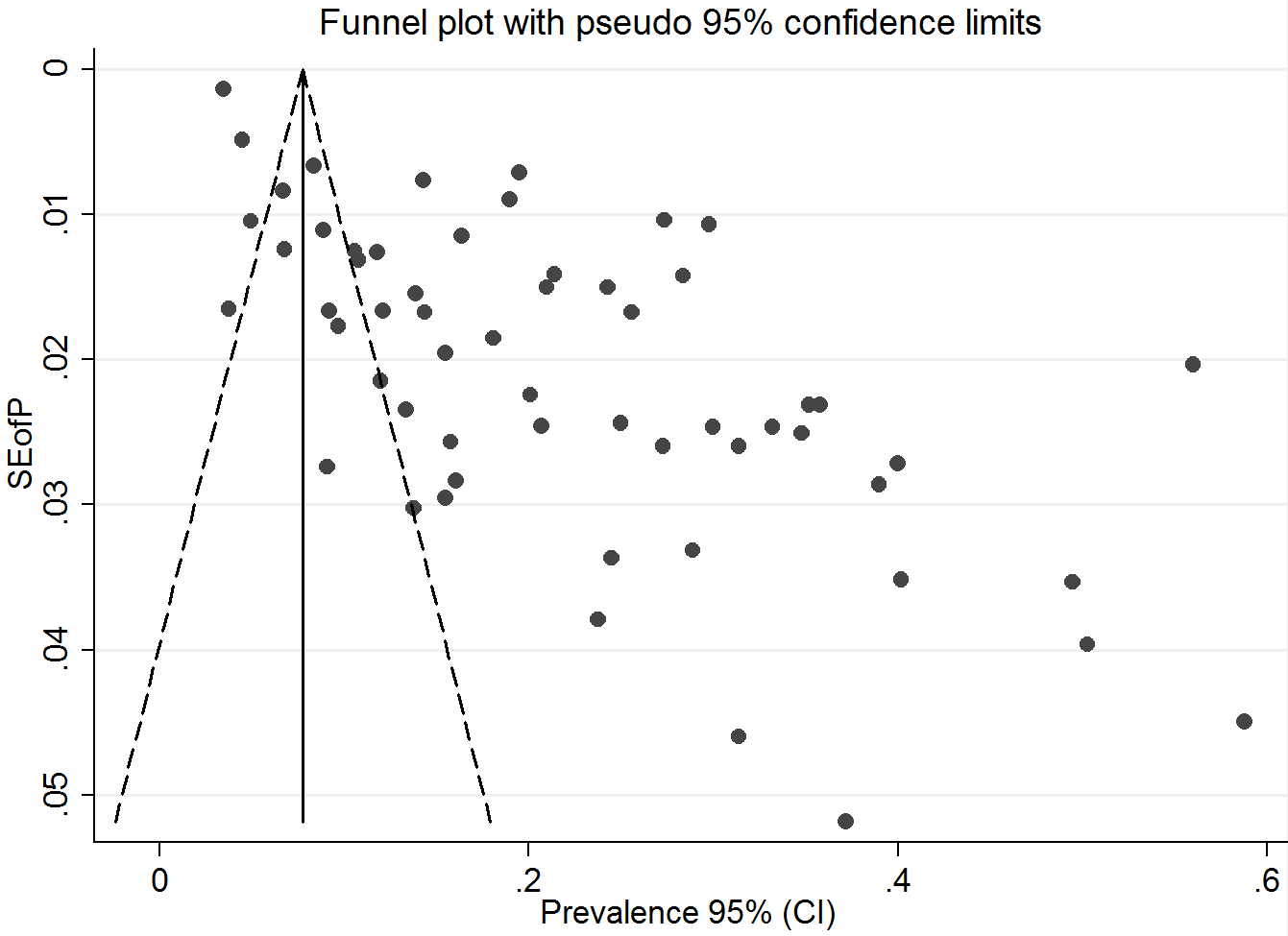


## Fig 3: Funnel plot

**
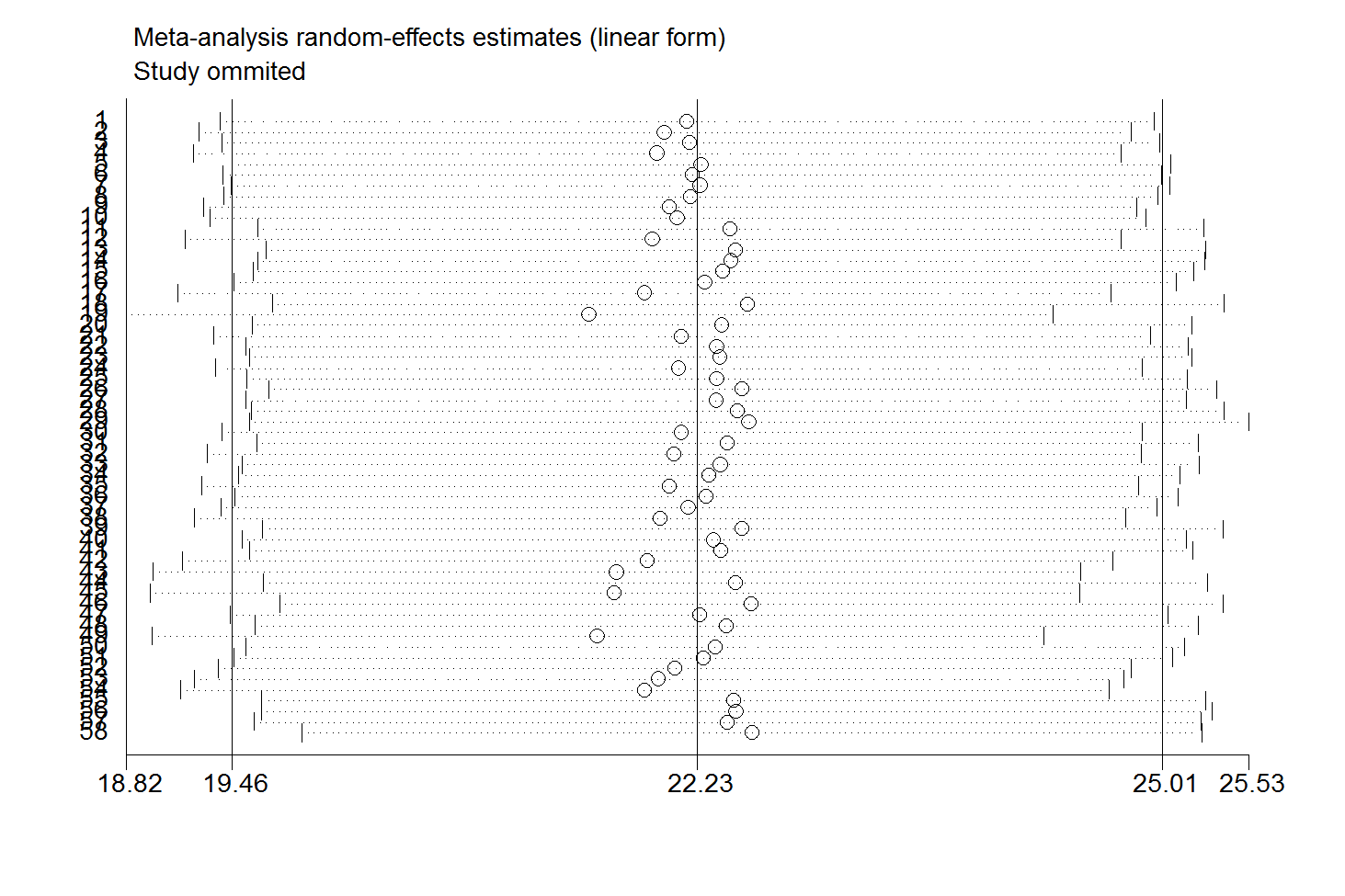
**

# Fig 3: Sensitivity analysis for postnatal depression prevalence estimates

# **Risk factors for postnatal depression**


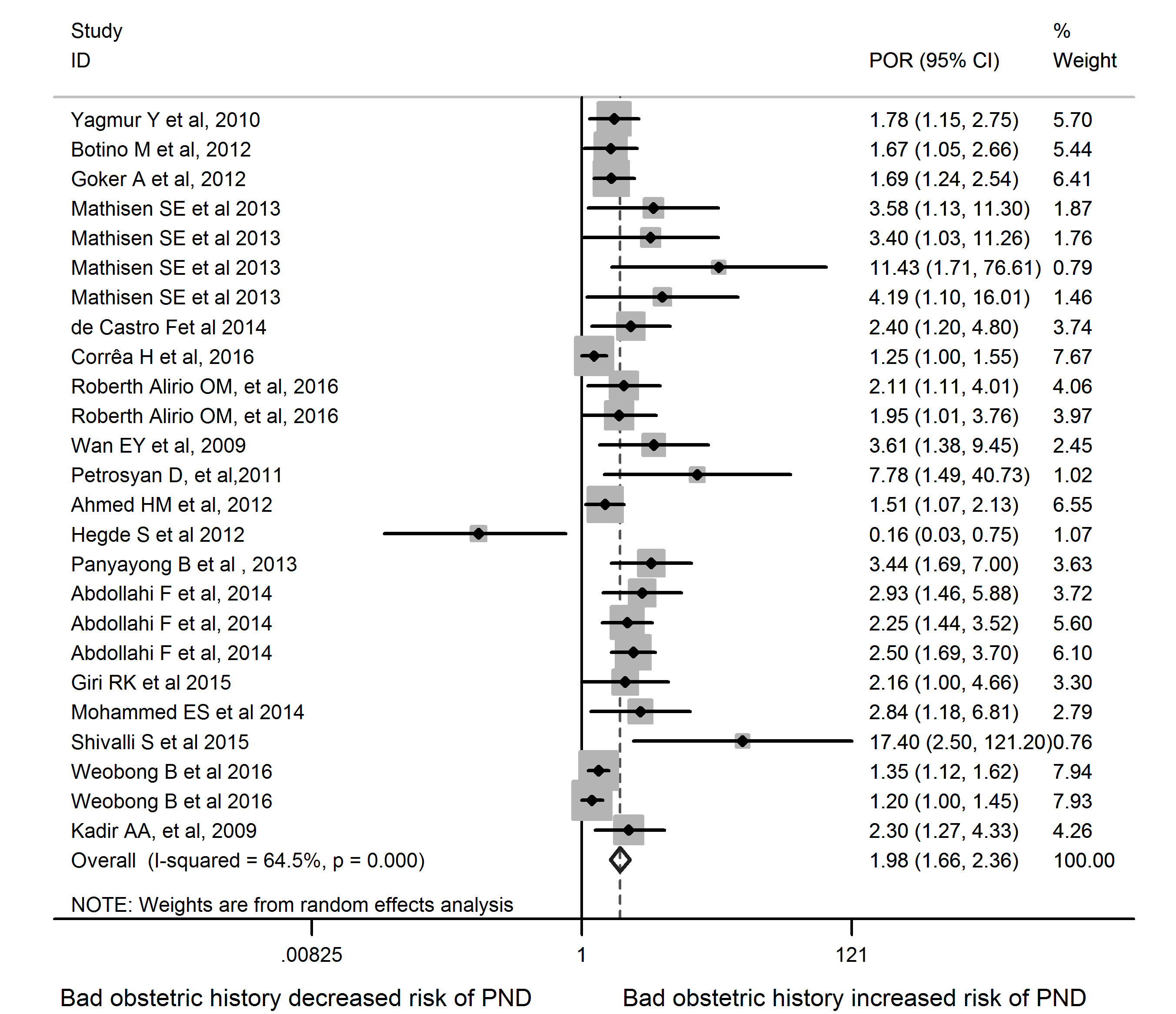


## Fig 4: Bad obstetric (*unplanned pregnancy, GDM, GHP, labor complication, history of emesis, multiparty*) history as a factor for postnatal depression


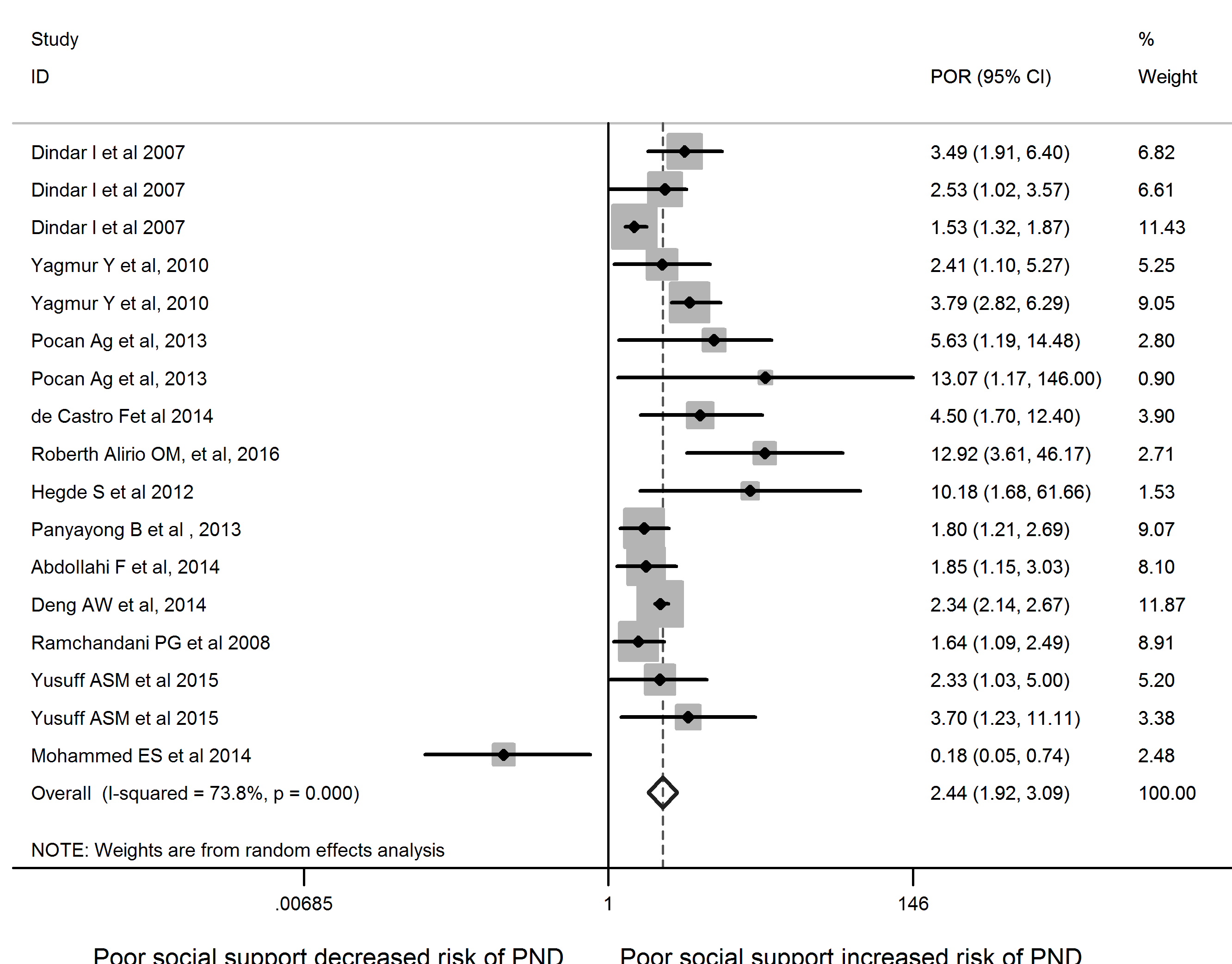


## Fig 5: Poor social support as a risk factor for postnatal depression


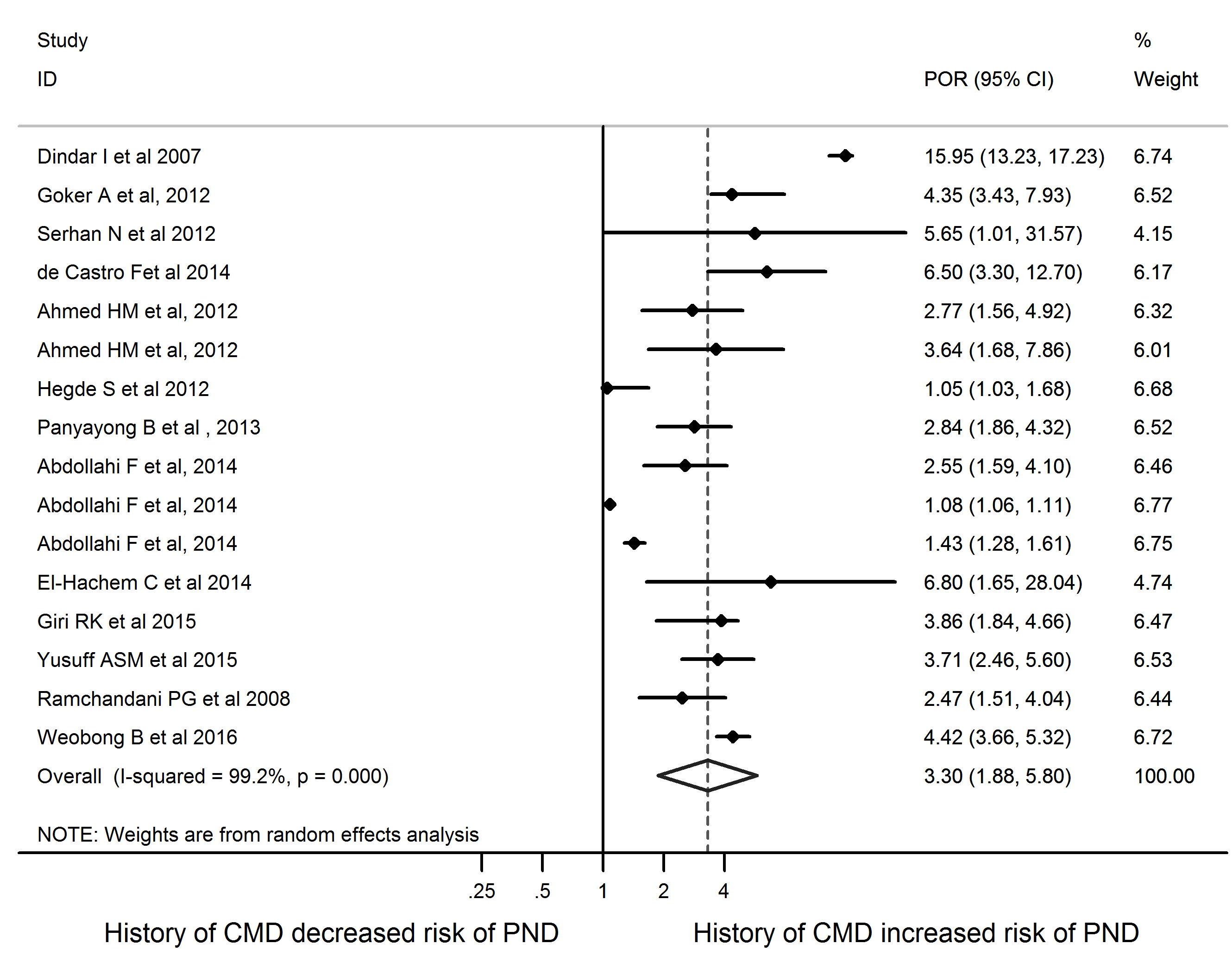


## Fig 6: History of common mental disorder (anxiety, depression, stress) as a risk factor for postnatal depression


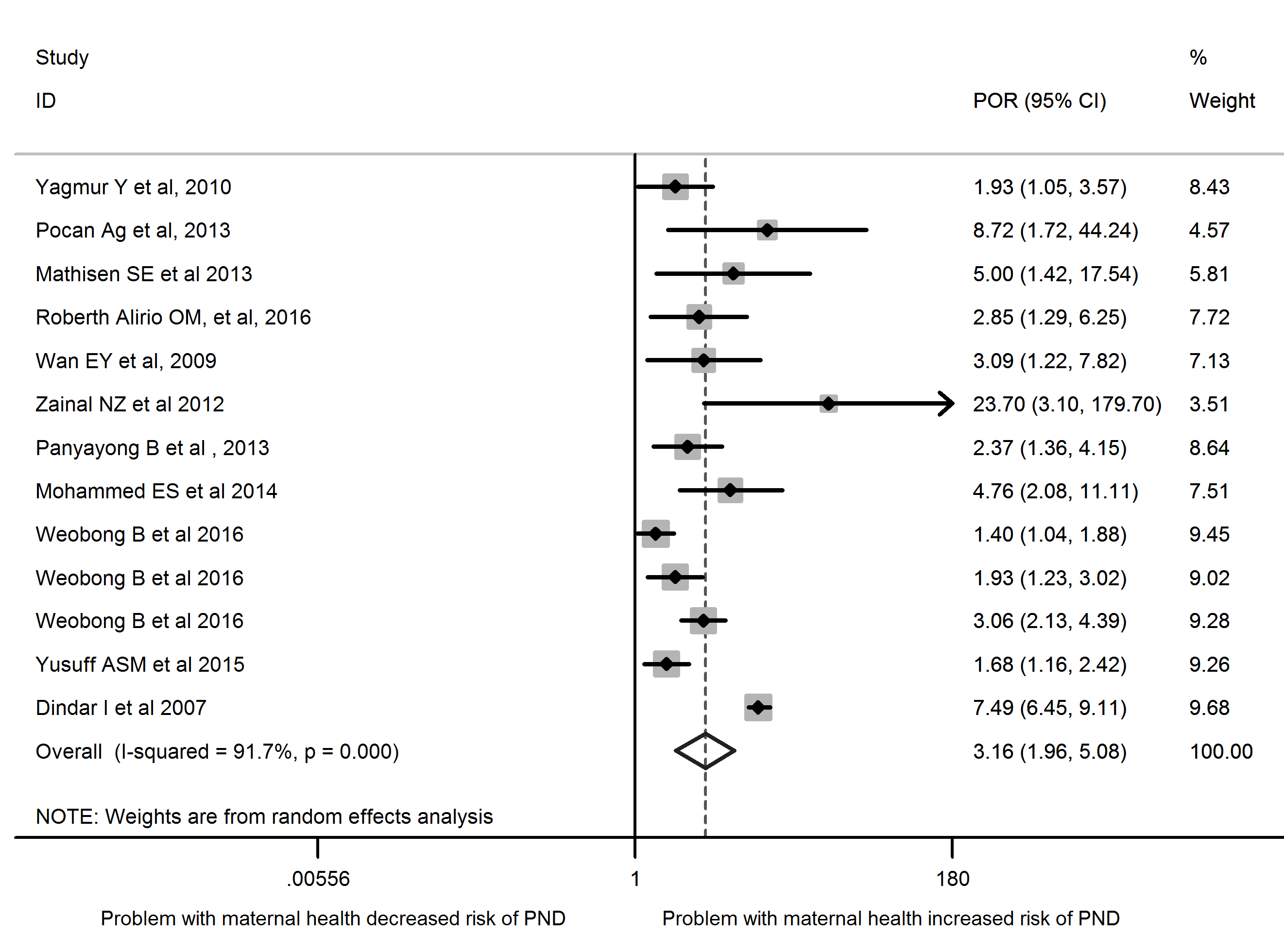


## Fig 7: Maternal and new born ill health as a risk factor for postnatal depression


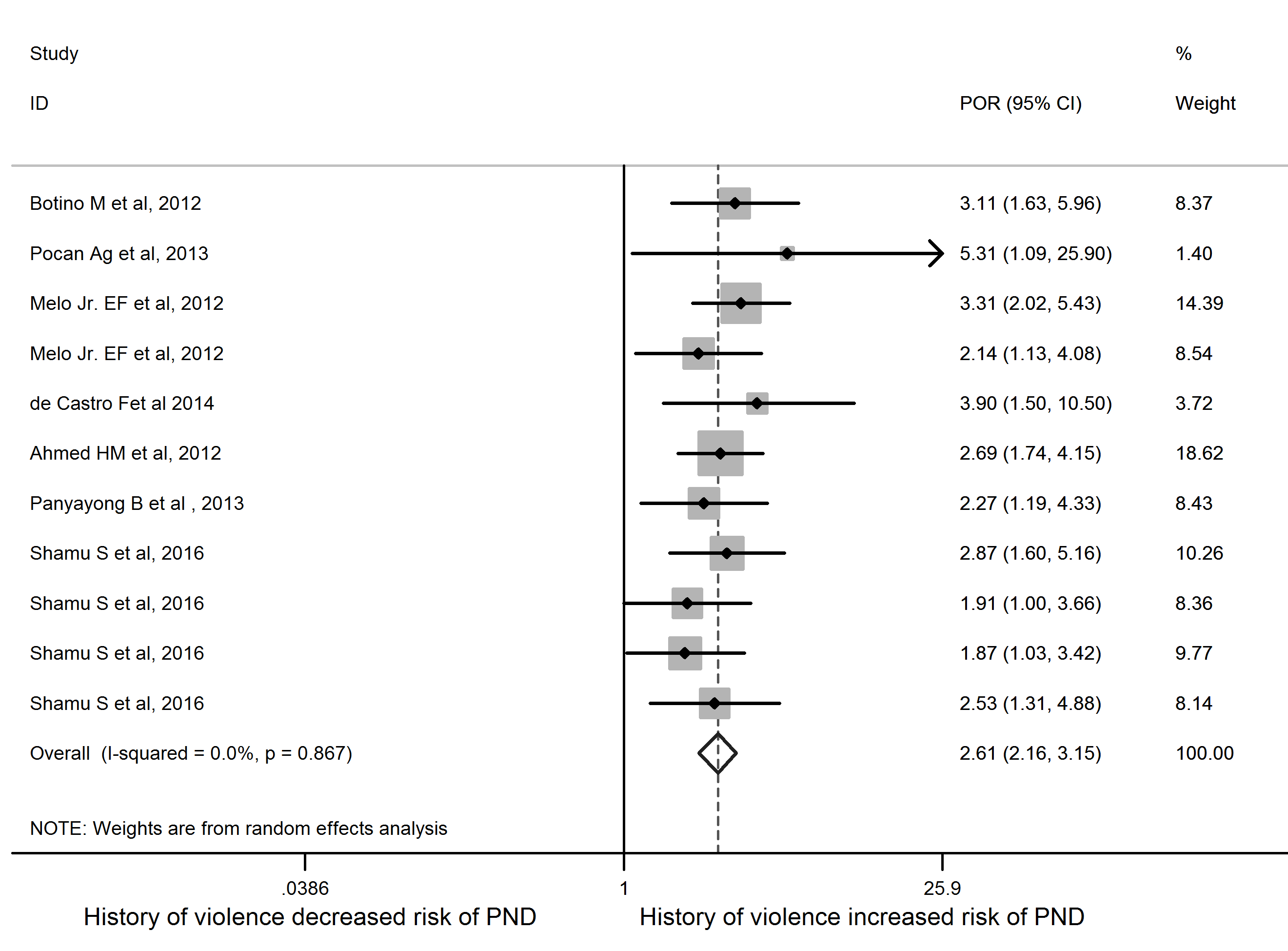


## Fig 8: Exposure to any form of life time violence as a risk factor for postnatal depression


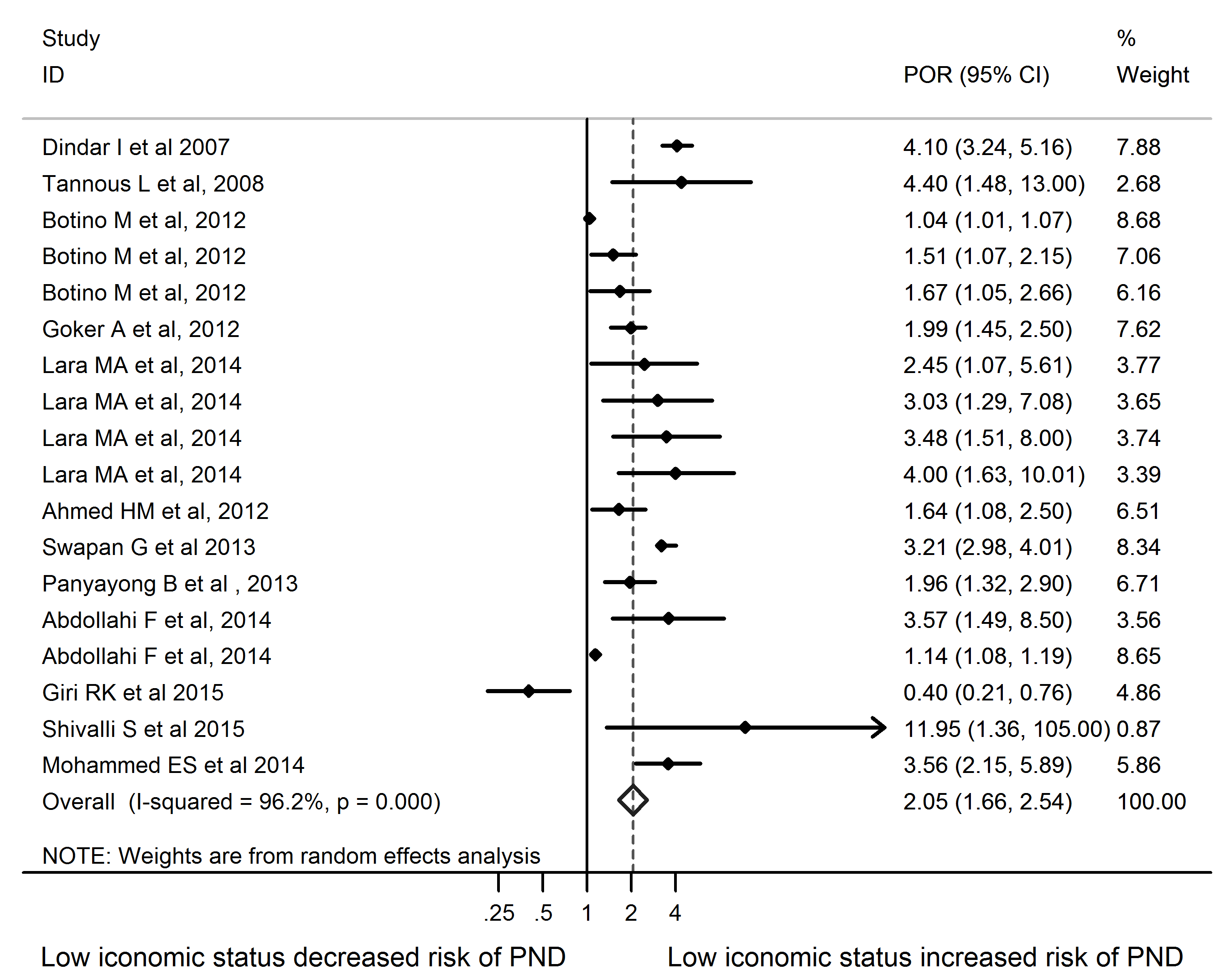


## Fig 9: Low economic status as a risk factor for postnatal depression


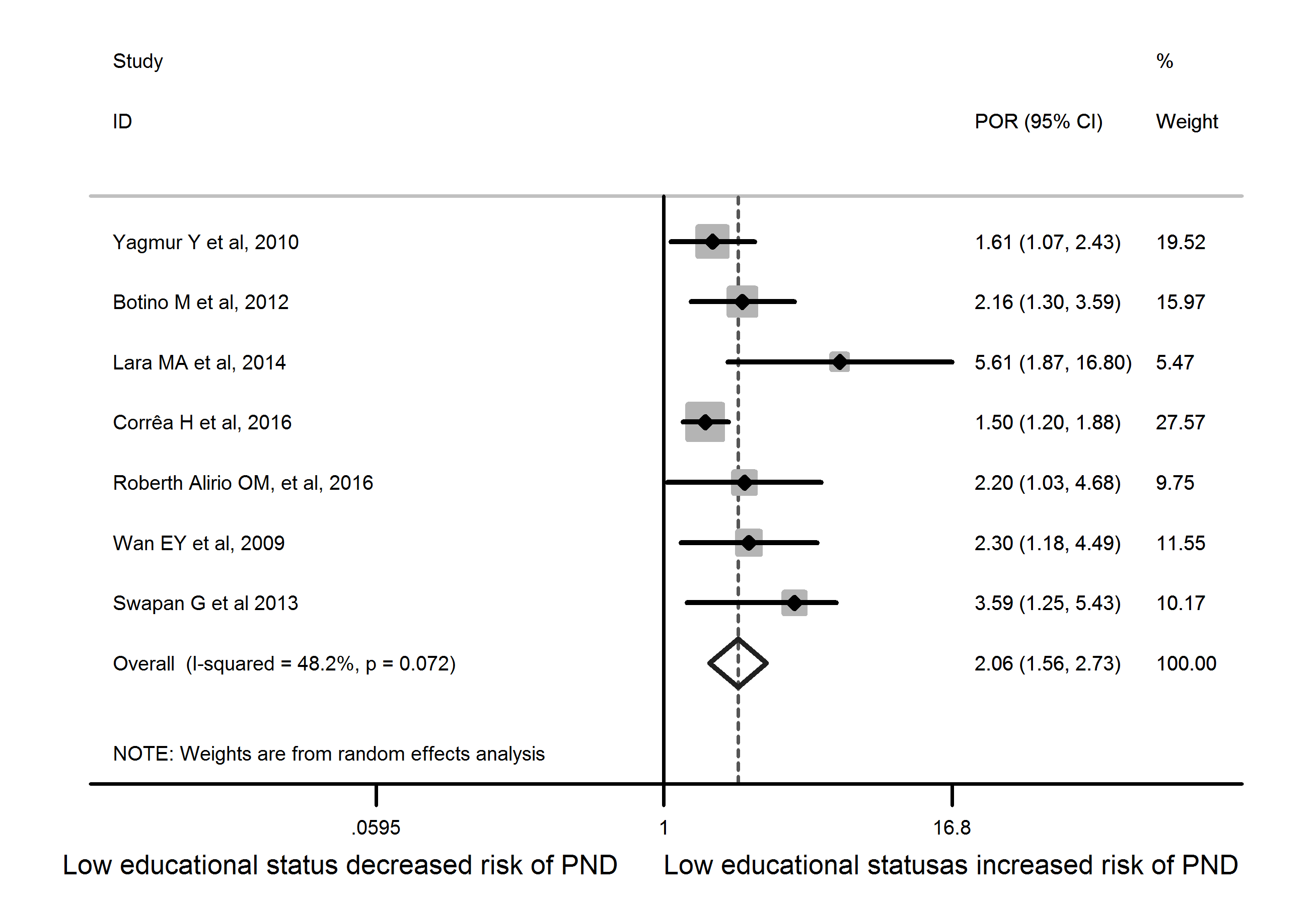


## Fig 10: Low educational status as a risk factor for postnatal depression

# **Chapter II: Postnatal depression and adverse infant health outcome**

## Table 2.2: Trim and fill analysis for postnatal depression effect on adverse infant health outcome

Filled

Meta-analysis (exponential form)

Method | Pooled 95% CI Asymptotic No. of studies

| Est Lower Upper z_value p_value 17

Fixed 1.189 1.144 1.236 8.806 0.000

Random 1.314 1.166 1.480 4.493 0.000

Test for heterogeneity: Q= 275.421 on 42 degrees of freedom (p= 0.000)

Moment-based estimate of between studies variance = 0.097

## Fig 2.1: Forest plot after trim and fill analysis (showed 10 studies left unpublished)


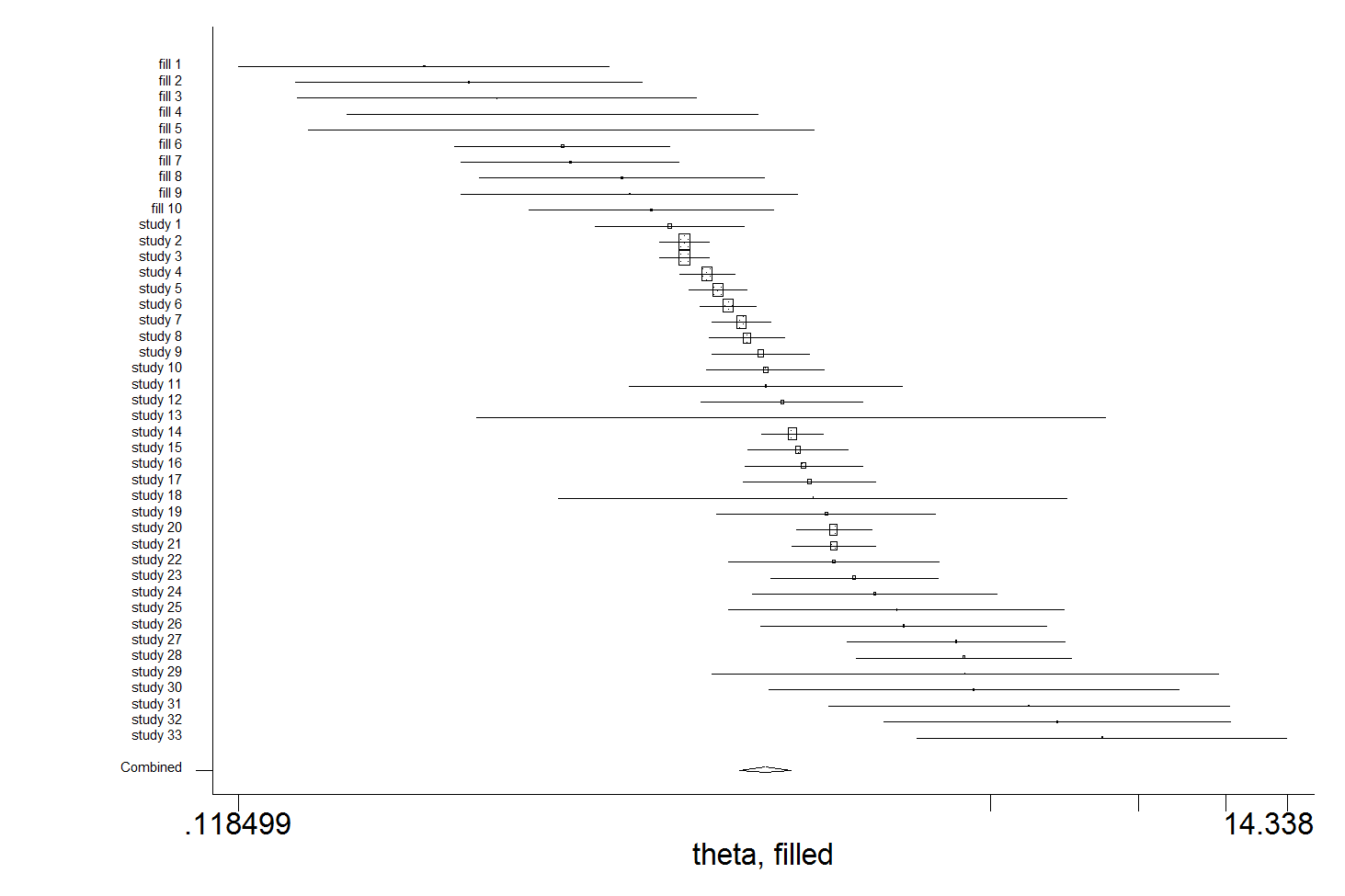


# Sub Analysis of effect of postnatal depression on adverse infant health outcomes


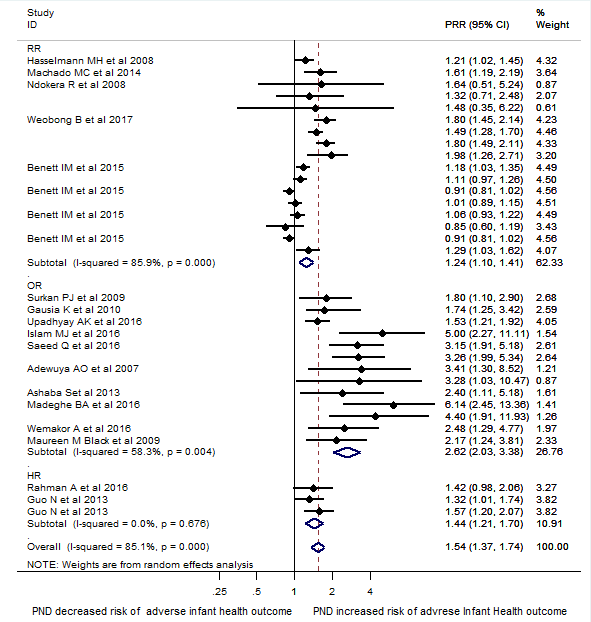


Fig 2.4: Effect of postnatal depression on adverse infant health outcome sub-analyzed by measure of association used in primary studies


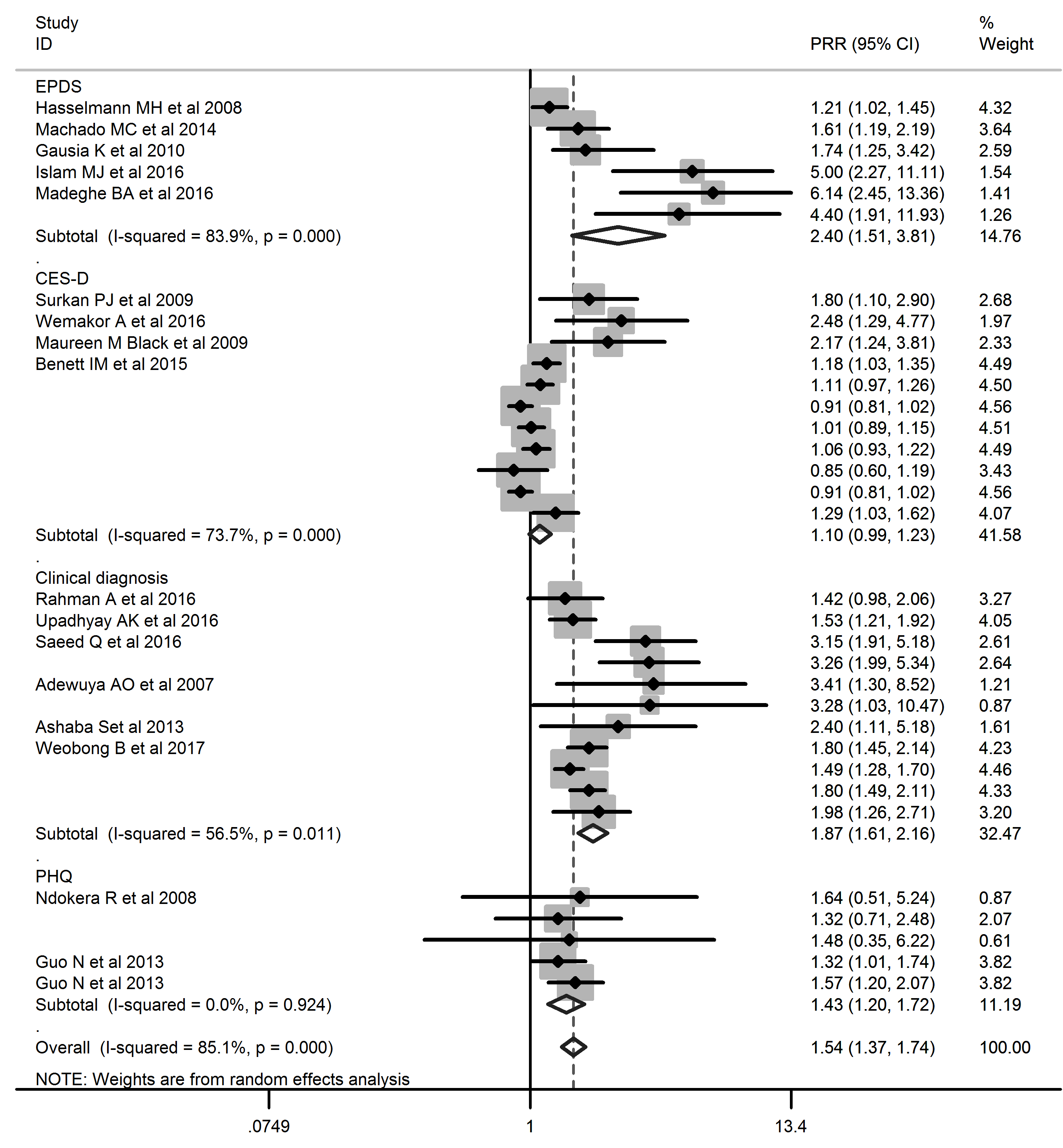


PND decreased the risk of adverse infant health outcome PND increased the risk of adverse infant health outcome

Fig 2.5: Effect of postnatal depression on adverse infant health outcome sub-analyzed by tool used for screening depression


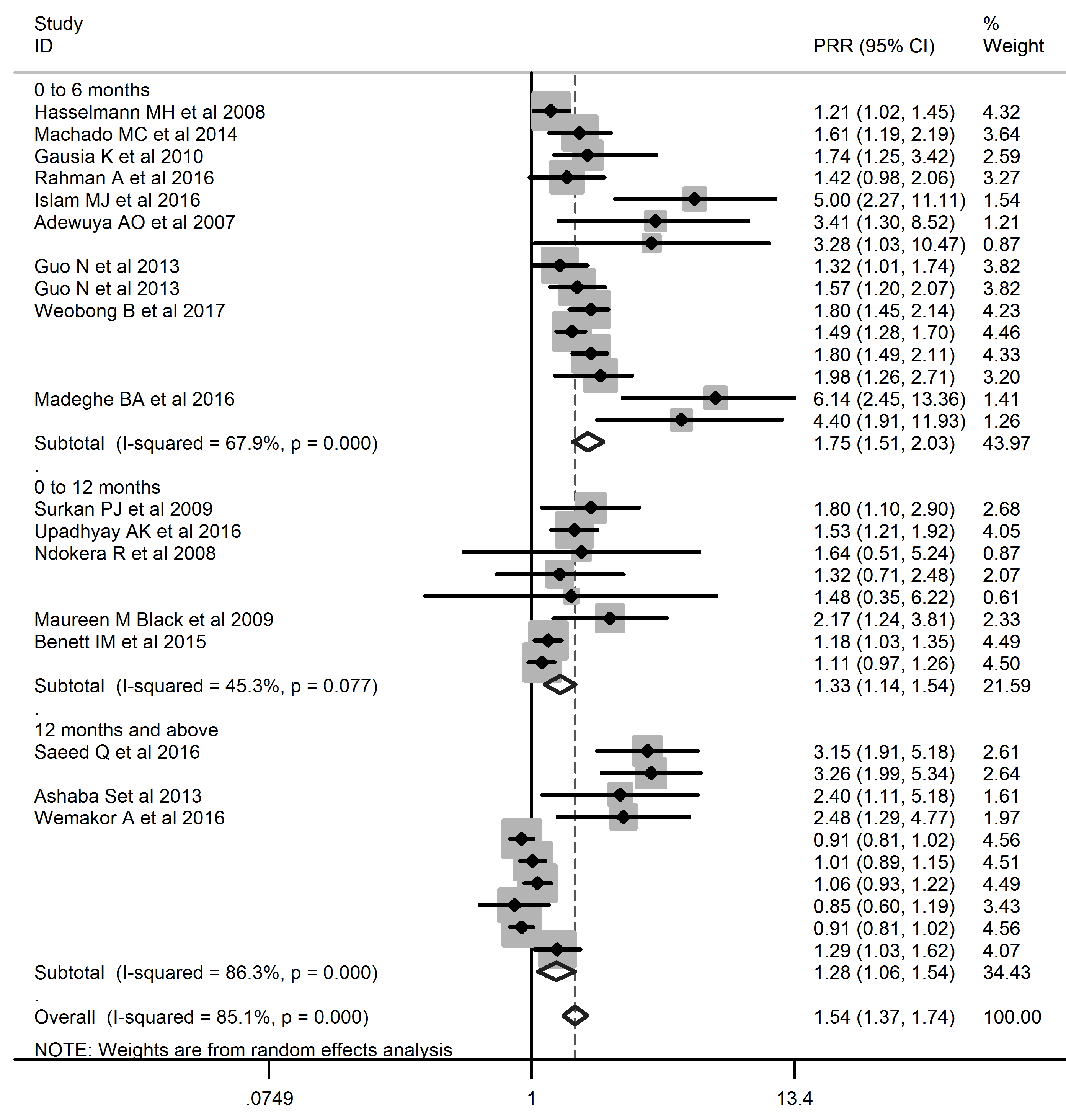


PND decreased the risk of adverse infant health outcome PND increased the risk of adverse infant health outcome

Fig 2.6: Effect of postnatal depression on adverse infant health outcome sub-analyzed by age of the infant


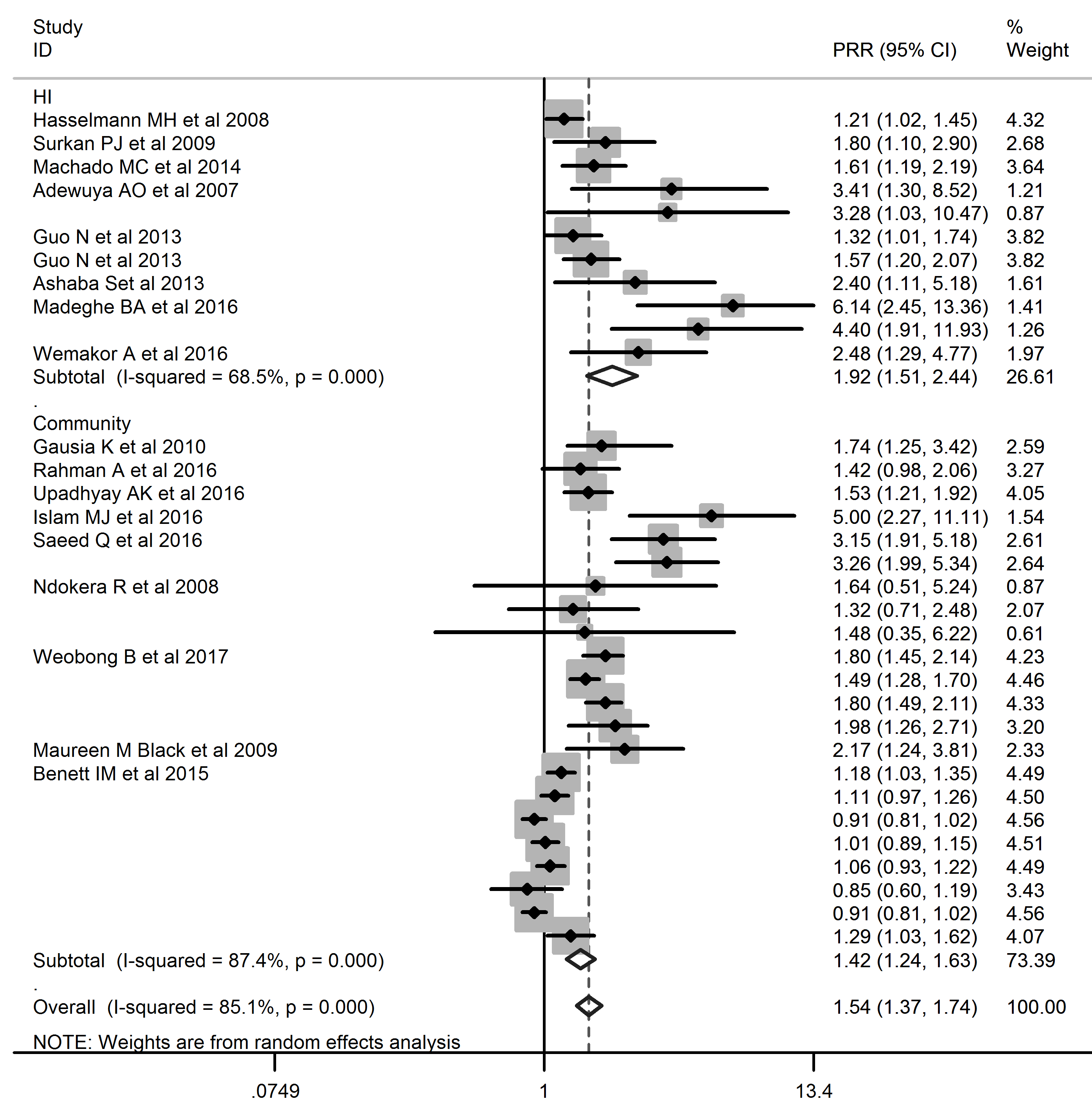


PND decreased the risk of adverse infant health outcome PND increased the risk of adverse infant health outcome

Fig 2.7. Effect of postnatal depression on adverse infant health outcome sub-analyzed by study setting


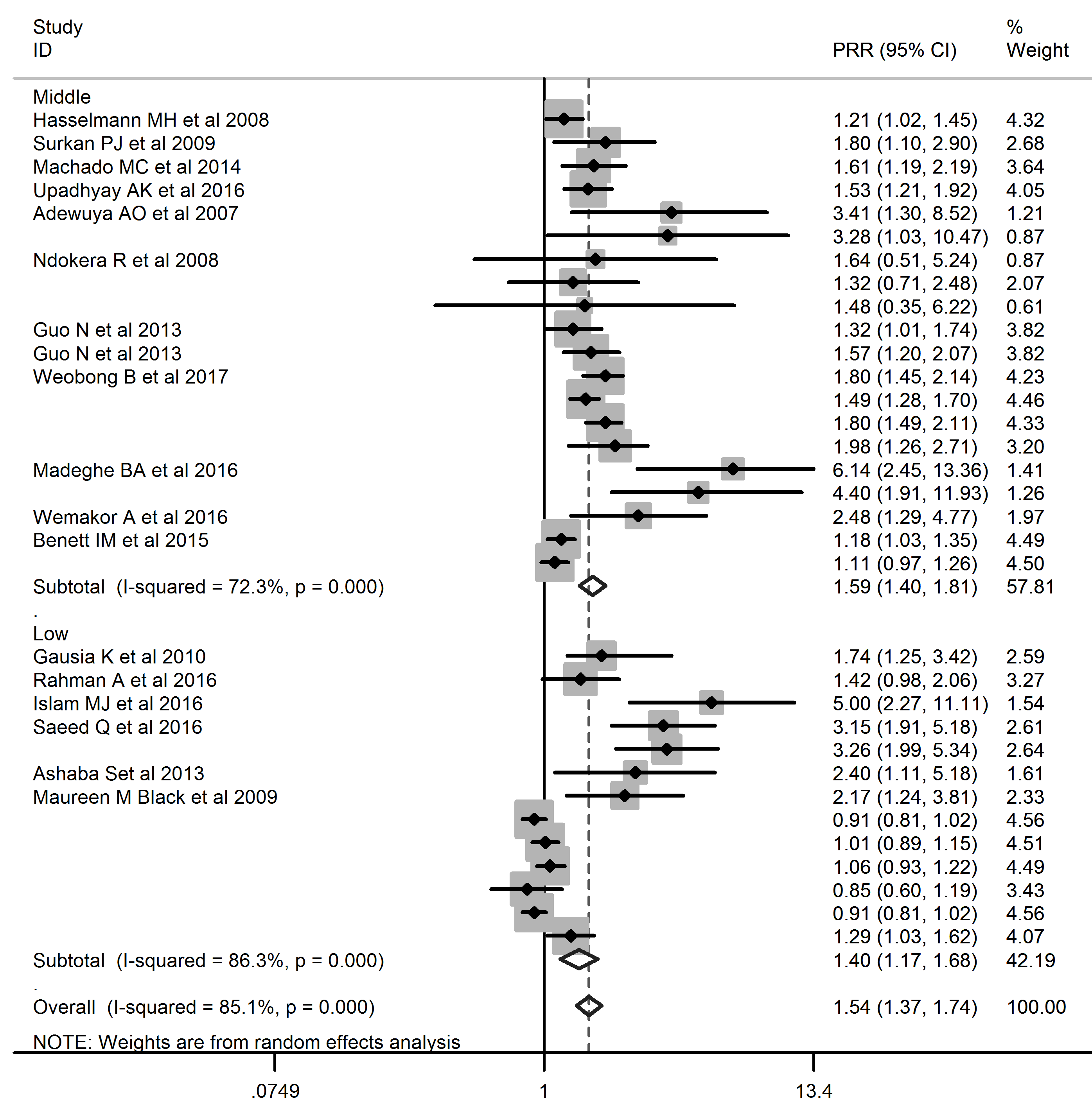


PND decreased the risk of adverse infant health outcome PND increased the risk of adverse infant health outcome

Fig 2.8. Effect of postnatal depression on adverse infant health outcomes sub-analyzed by country income


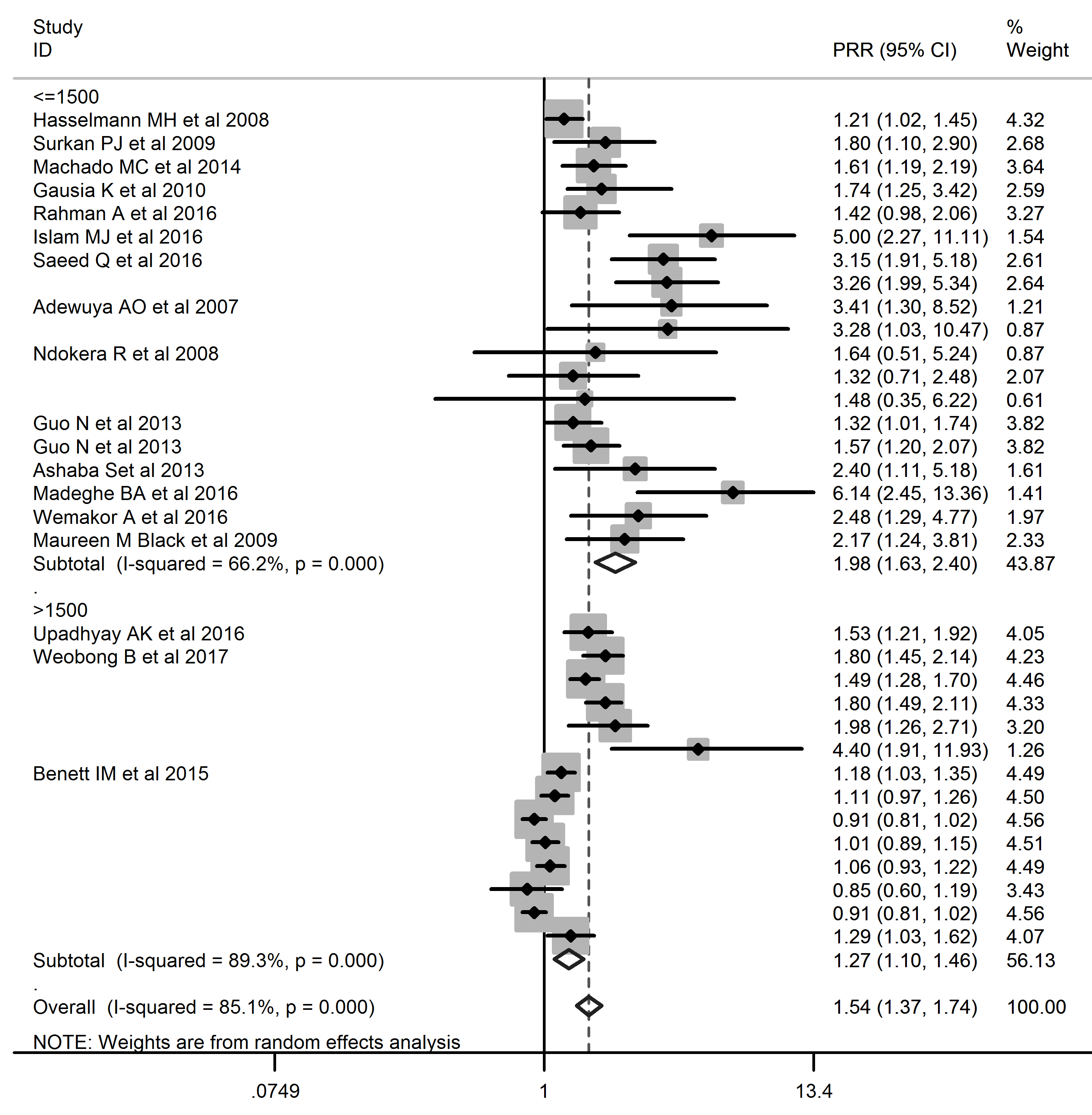


PND decreased the risk of adverse infant health outcome PND increased the risk of adverse infant health outcome

Fig 2.9: Effect of postnatal depression on adverse infant health outcomes sub-analyzed by sample size

1. Risk and protective factors associated with postnatal depression in Mexican adolescents. Journal of Psychosomatic Obstetrics & Gynecology. 2011;32(4):210-7.

2. Piyasil V, Pichaiyut P. Postpartum depression in the mothers of preterm infants at Queen Sirikit National Institute of Child Health. Journal of the Medical Association of Thailand = Chotmaihet thangphaet. 2011;94 Suppl 3:S91-4.

3. Chi X, Zhang P, Wu H, Wang J. Screening for postpartum depression and associated factors among women in China: A cross-sectional study. Frontiers in Psychology Vol 7 2016, ArtID 1668. 2016;7.

4. Dow A, Dube Q, Pence BW, Van Rie A. Postpartum depression and HIV infection among women in Malawi. Journal of acquired immune deficiency syndromes (1999). 2014;65(3):359-65.

5. Ganann R, Sword W, Thabane L, Newbold B, Black M. Predictors of Postpartum Depression Among Immigrant Women in the Year After Childbirth. 2016.

6. Garfield L, Holditch-Davis D, Carter CS, McFarlin BL, Schwertz D, Seng JS, et al. Risk factors for postpartum depressive symptoms in low-income women with very low-birth-weight infants. Advances in Neonatal Care (Lippincott Williams & Wilkins). 2015;15(1):E3-8.

7. Grote NK, Bledsoe SE. Predicting postpartum depressive symptoms in new mothers: the role of optimism and stress frequency during pregnancy. Health & Social Work. 2007;32(2):107-18.

8. Kossakowska-Petrycka K, Walecka-Matyja K. [Psychological causative factors in postpartum depression amongst women with normal and high-risk pregnancies]. 2007.

9. Krause KM, Østbye T, Swamy GK. Occurrence and correlates of postpartum depression in overweight and obese women: results from the Active Mothers Postpartum (AMP) study. Maternal & Child Health Journal. 2009;13(6):832-8.

10. Lovlie AL, Madar AA. Postpartum Depression Among Somali Women in Norway. 2017.

11. Molero KL, Urdaneta Machado JR, V C, Baabel Zambrano N, Contreras Benítez A, Azuaje Quiroz E, et al. Prevalencia de depresión posparto en puérperas adolescentes y adultas. Revista chilena de obstetricia y ginecología. 2014;79:294-304.

12. Nishigori H, Sugawara J, Obara T, Nishigori T, Sato K, Sugiyama T, et al. Surveys of postpartum depression in Miyagi, Japan, after the Great East Japan Earthquake. 2014.

13. Sharifi F, Nouraei S, Shahverdi E. The Relation of Pre and Postnatal Depression and Anxiety with Exclusive Breastfeeding. 2016.

14. Yehia DB, Callister LC, Hamdan-Mansour A. Prevalence and predictors of postpartum depression among Arabic Muslim Jordanian women serving in the military. 2013.

15. Lefkowitz DS, Baxt C, Evans JR. Prevalence and Correlates of Posttraumatic Stress and Postpartum Depression in Parents of Infants in the Neonatal Intensive Care Unit (NICU). Journal of Clinical Psychology in Medical Settings. 2010;17(3):230-7.

16. Sylven SM, Thomopoulos TP, Kollia N, Jonsson M, Skalkidou A. Correlates of postpartum depression in first time mothers without previous psychiatric contact. European psychiatry : the journal of the Association of European Psychiatrists. 2017;40:4-12.

17. Mohammad KI, Gamble J, Creedy DK. Prevalence and factors associated with the development of antenatal and postnatal depression among Jordanian women. Midwifery. 2011;27(6):e238-45.

18. Bodhare TN, Sethi P, Bele SD, Gayatri D, Vivekanand A. Postnatal quality of life, depressive symptoms, and social support among women in southern India. Women & Health. 2015;55(3):353-65.

19. El-Ibiary SY, Hamilton SP, Abel R, Erdman CA, Robertson PA, Finley PR. A pilot study evaluating genetic and environmental factors for postpartum depression. Innovations in Clinical Neuroscience. 2013;10(9-10):15-22.

20. Clarke K, Saville N, Shrestha B, Costello A, King M, Manandhar D, et al. Predictors of psychological distress among postnatal mothers in rural Nepal: a cross-sectional community-based study. J Affect Disord. 2014;156:76-86.

21. Garcia-Flores JR, Peregalli S, Garrido S, Garcia-Diaz S, Espada M, Sainz De La Cuesta R. Immediate postpartum evaluation of clinical markers for depression symptoms by means of the Edinburgh Postnatal Depression Scale. 2016.

22. Juliet Lakra A, Thomas S. Identify Risk Factors for Postnatal Depression among Antenatal Mothers - A Hospital Based Study. International Journal of Nursing Education. 2014;6(1):100-3.

23. Kabir ZN, Nasreen HE, Edhborg M. Intimate partner violence and its association with maternal depressive symptoms 6-8 months after childbirth in rural Bangladesh. Global health action. 2014;7:24725.

24. Kurth E. [Postnatal infant crying and maternal tiredness: examining their evolution and interaction in the first 12 weeks postpartum]. 2011.

25. Lakra AJ, Thomas S. Identify risk factors for postnatal depression among antenatal mothers - A hospital based study: International Journal of Nursing Education. 6 (1) (pp 100-103), 2014. Date of Publication: 2014.; 2014.

26. Lemola S, Stadlmayr W, Grob A. Infant irritability: The impact of fetal alcohol exposure, maternal depressive symptoms, and low emotional support from the husband: Infant Mental Health Journal. 30 (1) (pp 57-81), 2009. Date of Publication: 2009.; 2009.

27. Mao Q, Zhu LX, Su XY. A comparison of postnatal depression and related factors between Chinese new mothers and fathers. J Clin Nurs. 2011;20(5-6):645-52.

28. Meena PS, Soni R, Jain M, Jilowa CS, Omprakash. Cognitive Dysfunction and Associated Behaviour Problems in Postpartum Women: A Study from North India. East Asian Archives of Psychiatry. 2016;26(3):104-8.

29. Rahman A, Creed F. Outcome of prenatal depression and risk factors associated with persistence in the first postnatal year: Prospective study from Rawalpindi, Pakistan. Journal of Affective Disorders. 2007;100(1–3):115-21.

30. Smith-Nielsen J, Steele H, Mehlhase H, Cordes K, Steele M, Harder S, et al. Links Among High EPDS Scores, State of Mind Regarding Attachment, and Symptoms of Personality Disorder. Journal of Personality Disorders. 2015;29(6):771-93.

31. Tavares D, Quevedo L, Jansen K, Souza L, Pinheiro R, Silva R. Prevalence of suicide risk and comorbidities in postpartum women in Pelotas. Revista Brasileira de Psiquiatria. 2012;34:270-6.

32. Figueroa-Leigh F, Rojas P, Castanon C. Screening for postpartum depression in a private health care network in Chile. Family practice. 2015;32(4):431-5.

33. Hassert S, Kurpius SE, Tracey TJ. Testing a Conceptual Model of Postpartum Depressive Symptoms in the First Year. 2015.

34. Hung C-H, Lin C-J, Stocker J, Yu C-Y. Predictors of postpartum stress. Journal of Clinical Nursing. 2011;20(5-6):666-74.

35. Vliegen N, Luyten P, Besser A, Casalin S, Kempke S, Tang E. Stability and change in levels of depression and personality: a follow-up study of postpartum depressed mothers that were hospitalized in a mother-infant unit. Journal of Nervous & Mental Disease. 2010;198(1):45-51.

36. Yamamoto N, Abe Y, Arima K, Nishimura T, Akahoshi E, Oishi K, et al. Mental health problems and influencing factors in Japanese women 4 months after delivery. 2014.

37. O’Hara MW, Segre LS. Psychological disorders of pregnancy and the post partum. In R.S. Gibbs, B.Y. Karlan, A.F. Haney, & I. Nygaard (Eds.), Danforth’s obstetrics and gynecology (10th ed.). Philadelphia: Lippincott, Williams & Wilkins. 2008.

38. Feldman R, Granat A, Pariente C, Kanety H, Kuint J, Gilboa-Schechtman E. Maternal depression and anxiety across the postpartum year and infant social engagement, fear regulation, and stress reactivity. Journal of the American Academy of Child and Adolescent Psychiatry. 2009;48(9):919-27.

39. Dois A, Uribe C, Villarroel L, Contreras A. Factores de riesgo asociados a síntomas depresivos post parto en mujeres de bajo riesgo obstétrico atendidas en el sistema público. Revista médica de Chile. 2012;140:719-25.

40. Cheng CY, Pickler RH. Perinatal stress, fatigue, depressive symptoms, and immune modulation in late pregnancy and one month postpartum. 2014.

41. Kim Y. The self-care ability for health practices in rural Hispanic women experiencing depressive symptoms during postpartum: Azusa Pacific University; 2014.

42. Nash CO. The relationship between sleep quality, physical activity and postpartum mood. Dissertation Abstracts International: Section B: The Sciences and Engineering. 2016;76(8-B(E)):No Pagination Specified.

43. O'Hara MW, Wisner KL. Perinatal mental illness: definition, description and aetiology. Best practice & research Clinical obstetrics & gynaecology. 2014;28(1):3-12.

44. O'Hara MW. Postpartum depression: what we know. Journal of Clinical Psychology. 2009;65(12):1258-69.

45. Ahmet GÜRhan P, ÖZlem Erden A, Ayse Huriye ParlakgÜMÜ S, ÇIĞDem G, Anil Barak D. THE INCIDENCE OF AND RISK FACTORS FOR POSTPARTUM DEPRESSION AT AN URBAN MATERNITY CLINIC IN TURKEY. International Journal of Psychiatry in Medicine. 2013;46(2):179-94.

46. Galeshi M, Mirghafourvand M, Abbasnezhad SM, Afsari A. Relationship between postpartum anxiety and depression and marital satisfaction. [Persian]: Journal of Mazandaran University of Medical Sciences. 25 (134) (pp 351-356), 2016. Date of Publication: 2016.; 2016.

47. Johnson AR, Edwin S, Joachim N, Mathew G, Ajay S, Joseph B. Postnatal depression among women availing maternal health services in a rural hospital in South India. Pakistan Journal of Medical Sciences. 2015;31(2):408-13.

48. Kakyo TA, Muliira JK, Mbalinda SN, Kizza IB, Muliira RS. Factors associated with depressive symptoms among postpartum mothers in a rural district in Uganda. Midwifery. 2012;28(3):374-9.

49. Kumwar D, Corey EK, Sharma P, Risal A. Screening for Postpartum Depression and Associated Factors among Women who Deliver at a University Hospital, Nepal. Kathmandu University medical journal (KUMJ). 2015;13(49):44-8.

50. Roomruangwong C, Withayavanitchai S, Maes M. Antenatal and postnatal risk factors of postpartum depression symptoms in Thai women: A case-control study: Sexual and Reproductive Healthcare. 10 (pp 25-31), 2016. Date of Publication: 01 Dec 2016.; 2016.

51. Suhitharan T, Pham TPT, Chen H, Assam PN, Sultana R, Han N-LR, et al. Investigating analgesic and psychological factors associated with risk of postpartum depression development: A case-control study. Neuropsychiatric Disease and Treatment Vol 12 2016, ArtID 1333-1339. 2016;12.

52. Avan B, Richter LM, Ramchandani PG, Norris SA, Stein A. Maternal postnatal depression and children's growth and behaviour during the early years of life: exploring the interaction between physical and mental health. Archives of Disease in Childhood. 2010;95(9):690-5.

53. Herba CM. Maternal depression and child behavioural outcomes. The Lancet Psychiatry. 2014;1(6):408-9.

54. Maselko J, Sikander S, Bangash O, Bhalotra S, Franz L, Ganga N, et al. Child mental health and maternal depression history in Pakistan. Social Psychiatry and Psychiatric Epidemiology. 2016;51(1):49-62.

55. Verkuijl NE, Richter L, Norris SA, Stein A, Avan B, Ramchandani PG. Postnatal depressive symptoms and child psychological development at 10 years: A prospective study of longitudinal data from the South African Birth to Twenty cohort. The Lancet Psychiatry. 2014;1(6):454-60.

56. Zhu P, Sun M-S, Hao J-H, Chen Y-J, Jiang X-M, Tao R-X, et al. Does prenatal maternal stress impair cognitive development and alter temperament characteristics in toddlers with healthy birth outcomes? Developmental Medicine & Child Neurology. 2014;56(3):283-9.

57. Cavalcante-Neto JL, de Paula CS, Florêncio TMMT, de Miranda CT. Disability due to maternal common mental disorders (CMDs) as a risk factor for chronic childhood malnutrition: Cross-sectional study. Sao Paulo Medical Journal. 2016;134(3):228-33.

58. Choi KW, Sikkema KJ, Vythilingum B, Geerts L, Faure SC, Watt MH, et al. Maternal childhood trauma, postpartum depression, and infant outcomes: Avoidant affective processing as a potential mechanism. Journal of Affective Disorders. 2017;211:107-15.

59. Manzolli P, Nunes MA, Schmidt MI, Ferri CP. Abuse against women, depression, and infant morbidity: a primary care cohort study in Brazil. American journal of preventive medicine. 2012;43(2):188-95.

60. Rondó PHC, Rezende G, Lemos JO, Pereira JA. Maternal stress and distress and child nutritional status. European Journal of Clinical Nutrition. 2013;67(4):348-52.

61. Chong S-C, Broekman BF, Qiu A, Aris IM, Chan YH, Rifkin-Graboi A, et al. Anxiety and depression during pregnancy and temperament in early infancy: Findings from a multi-ethnic, Asian, prospective birth cohort study. Infant Mental Health Journal. 2016;37(5):584-98.

62. Decaro JA, Manyama M, Wilson W. Household-level predictors of maternal mental health and systemic inflammation among infants in Mwanza, Tanzania. American Journal of Human Biology. 2016;28(4):461-70.

63. Lukose A, Ramthal A, Thomas T, Bosch R, Kurpad AV, Duggan C, et al. Nutritional factors associated with antenatal depressive symptoms in the early stage of pregnancy among Urban South Indian women. Maternal and Child Health Journal. 2014;18(1):161-70.

64. Motlhatlhedi K, Setlhare V, Ganiyu AB, Firth JA. Association between depression in carers and malnutrition in children aged 6 months to 5 years. African Journal of Primary Health Care and Family Medicine. 2017;9(1).

65. Nahar B, Hossain I, Hamadani JD, Ahmed T, Grantham-Mcgregor S, Persson LA. Effect of a food supplementation and psychosocial stimulation trial for severely malnourished children on the level of maternal depressive symptoms in Bangladesh. Child: Care, Health and Development. 2015;41(3):483-93.

66. Otake Y, Nakajima S, Uno A, Kato S, Sasaki S, Yoshioka E, et al. Association between maternal antenatal depression and infant development: a hospital-based prospective cohort study. Environmental Health & Preventive Medicine. 2014;19(1):30-45.

67. Sikander S, Maselko J, Zafar S, Haq Z, Ahmad I, Ahmad M, et al. Cognitive-behavioral counseling for exclusive breastfeeding in rural pediatrics: A cluster RCT. Pediatrics. 2015;135(2):e424-e31.

68. Vedova A. Maternal psychological state and infant's temperament at three months. Journal of Reproductive and Infant Psychology. 2014;32(5):520-34.

69. Yoo JP, Chung GH, Lee SG. The Associations Between Socioeconomic Status, Caregivers' Depressive Symptoms, Children's Health-Promoting Behavior, and Children's Physical Health: A Mediation Model. Asian Social Work and Policy Review. 2015;9(2):177-93.

70. Heerman WJ, Taylor JL, Wallston KA, Barkin SL. Parenting Self-Efficacy, Parent Depression, and Healthy Childhood Behaviors in a Low-Income Minority Population: A Cross-Sectional Analysis. Maternal and Child Health Journal. 2017;21(5):1156-65.

71. Jafree SR, Zakar R, Zakar MZ. Factors Associated with Low Birth Weight of Children Among Employed Mothers in Pakistan. Maternal and Child Health Journal. 2015;19(9):1993-2002.

72. Kaaya S, Garcia ME, Li N, Lienert J, Twayigize W, Spiegelman D, et al. Association of maternal depression and infant nutritional status among women living with HIV in Tanzania. Maternal and Child Nutrition. 2016;12(3):603-13.

73. Okronipa HE, Marquis GS, Lartey A, Brakohiapa L, Perez-Escamilla R, Mazur RE. Postnatal depression symptoms are associated with increased diarrhea among infants of HIV-positive Ghanaian mothers. AIDS and behavior. 2012;16(8):2216-25.

74. Perosa GB, Canavez IC, Silveira FCP, Padovani FHP, Peraçoli JC. Depressive and anxious symptoms in mothers of newborns with and without malformations. Revista Brasileira de Ginecologia e Obstetricia. 2009;31(9):433-9.

75. Flores-Quijano ME, Cordova A, Contreras-Ramirez V, Farias-Hernandez L, Cruz Tolentino M, Casanueva E. Risk for postpartum depression, breastfeeding practices, and mammary gland permeability. Journal of human lactation : official journal of International Lactation Consultant Association. 2008;24(1):50-7.
